# Impact of source tissue and *ex vivo* expansion on the characterization of goat mesenchymal stem cells

**DOI:** 10.1186/2049-1891-6-1

**Published:** 2015-01-11

**Authors:** Nuradilla Mohamad-Fauzi, Pablo J Ross, Elizabeth A Maga, James D Murray

**Affiliations:** Department of Animal Science, University of California, Davis, California 95616 USA; Department of Population Health and Reproduction, University of California, Davis, California 95616 USA; Institute of Ocean and Earth Sciences, University of Malaya, 50603 Kuala Lumpur, Malaysia

**Keywords:** Adipose, Bone marrow, Characterization, Differentiation, Goat, Mesenchymal stem cells

## Abstract

**Background:**

There is considerable interest in using goats as models for genetically engineering dairy animals and also for using stem cells as therapeutics for bone and cartilage repair. Mesenchymal stem cells (MSCs) have been isolated and characterized from various species, but are poorly characterized in goats.

**Results:**

Goat MSCs isolated from bone marrow (BM-MSCs) and adipose tissue (ASCs) have the ability to undergo osteogenic, adipogenic and chondrogenic differentiation. Cytochemical staining and gene expression analysis show that ASCs have a greater capacity for adipogenic differentiation compared to BM-MSCs and fibroblasts. Different methods of inducing adipogenesis also affect the extent and profile of adipogenic differentiation in MSCs. Goat fibroblasts were not capable of osteogenesis, hence distinguishing them from the MSCs. Goat MSCs and fibroblasts express CD90, CD105, CD73 but not CD45, and exhibit cytoplasmic localization of OCT4 protein. Goat MSCs can be stably transfected by Nucleofection, but, as evidenced by colony-forming efficiency (CFE), yield significantly different levels of progenitor cells that are robust enough to proliferate into colonies of integrants following G418 selection. BM-MSCs expanded over increasing passages *in vitro* maintained karyotypic stability up to 20 passages in culture, exhibited an increase in adipogenic differentiation and CFE, but showed altered morphology and amenability to genetic modification by selection.

**Conclusions:**

Our findings provide characterization information on goat MSCs, and show that there can be significant differences between MSCs isolated from different tissues and from within the same tissue. Fibroblasts do not exhibit trilineage differentiation potential at the same capacity as MSCs, making it a more reliable method for distinguishing MSCs from fibroblasts, compared to cell surface marker expression.

**Electronic supplementary material:**

The online version of this article (doi:10.1186/2049-1891-6-1) contains supplementary material, which is available to authorized users.

## Background

Mesenchymal stem cells (MSCs), also known as multipotent stromal cells, are one of the most studied adult stem cells for their ease of culture *ex vivo* and their multipotentiality, as well as supportive functions *in vivo*. Believed to reside in virtually all post-natal tissues [1], MSCs have been isolated and characterized from a variety of tissue types, most commonly from bone marrow [2, 3] and adipose tissue [4]. Even though MSCs isolated from different tissues appear similar in morphology and their ability to differentiate into osteogenic, chondrogenic and adipogenic lineages [5], they can display phenotypical differences, including in differentiation potential [6–9] and cell surface proteins [10, 11]. Bone marrow-derived MSCs (BM-MSCs) have a higher capacity to differentiate into osteogenic and chondrogenic lineages [6, 7], whereas adipose-derived MSCs (ASCs) are better at differentiating into adipocytes [8, 9]. The influence of source tissue, along with variations due to species, donor, and culture methods, have challenged the consistency of reports in the literature and complicated the understanding of MSC biology.

Goats are widely used as large animal models for bone tissue engineering as they have knee joints that are similar to humans [12]. Due to the osteogenic and chondrogenic potential of MSCs, goat MSCs are utilized in tissue engineering applications for bone and cartilage regeneration, such as repairing segmental bone defects [13, 14] and restoring articular cartilage [15, 16]. Some studies also employed genetic engineering of goat MSCs for the purpose of cell-based therapy, such as generating GFP-positive MSCs for tracking engraftment of transplanted cells [17], enhancing differentiation potential of transplanted MSCs via expression of transgenes [18] and expressing factors to facilitate the tissue generation process [19]. Reports on the characterization of goat MSCs are relatively more recent and amount to little compared to MSCs isolated from other more commonly used species such as the human, mouse and pig. Many of the reports utilizing goat MSCs, mostly from bone marrow, for tissue engineering lack characterization information. There are relatively few reports on goat MSCs isolated from other tissues, such as adipose tissue [20, 21] and umbilical cord [22, 23]. Little information exists on comparisons between goat MSCs isolated from different source tissues, and on comparisons with fibroblasts, which are morphologically similar to and share characteristics with MSCs [24, 25].

Adipogenic differentiation *in vitro* is easy to characterize, as distinct morphological changes that occur are easily visualized and molecular markers of adipogenesis are well-described. Exploring adipogenic differentiation of MSCs in culture may provide a window into understanding adipogenesis, especially upstream in the pathway where lineage commitment occurs. This cannot be studied in pre-adipocyte cell lines such as 3 T3-1 cells, which are already lineage-committed. Additionally, studying adipogenic differentiation in MSCs may have implications for meat animals such as goats and cows [26, 27], as intramuscular adipocyte differentiation is initiated by MSCs [28]. As goat MSCs are researched extensively for applications in bone and cartilage tissue regeneration, measuring adipogenesis may provide valuable information to inform the selection of MSC cultures for these applications.

For use in experiments and clinical applications, MSCs are needed in amounts that are larger than the starting population isolated from a sample and must be expanded *in vitro*. With increasing population doublings, studies have observed an increase in doubling time [29], increase in cellular senescence [30], changes in cell surface marker profile [31], and loss in multipotentiality [32, 33]. This suggests that *ex vivo* culture conditions for MSCs outside of their niches *in vivo* is not sufficient for maintaining MSC characteristics over long-term expansion. To date, changes in MSC characteristics due to long-term *ex vivo* culture have not yet been characterized in MSCs isolated from goats.

In this study, we report characterization of three lines of putative MSCs isolated from bone marrow and adipose tissue of neonatal kid goats. We provide a comparison between MSC lines isolated from the same tissue type as well as from different source tissues. Osteogenic, chondrogenic and adipogenic differentiation, as well as the expression of cell surface markers, were investigated. Adipogenic differentiation capacity was measured by both the extent of Oil Red O staining and mRNA expression of genes involved in adipogenesis. These characteristics also were compared to fibroblasts isolated from goat ear tissue. Using MSCs, we also assessed colony-forming efficiency, expression of pluripotency markers and transfection efficiency as well as integration of an introduced plasmid construct. BM-MSCs were also expanded up to 20 passages, and examined for their adipogenic differentiation, colony-forming efficiency, cell surface marker expression and potential for genetic modification.

## Methods

### Isolation and establishment of cell lines

Bone marrow and adipose tissue samples were collected from two male neonatal kid goats (9003 and 9004). MSCs were isolated using methods described in Monaco et al. [8]. Three lines were established: one bone marrow-derived line from individual 9004 (9004 BM-MSC), as well as one bone marrow- and one adipose-derived line from individual 9003 (9003 BM-MSC and 9003 ASC, respectively).

Ear fibroblasts (1014 EF) were isolated from a juvenile (2–3 months old) male goat from the UC Davis herd. A biopsy of the ear was taken and stored in PBS until it was processed. The outer skin was removed with a scalpel, and the remaining tissue was diced into approximately 3 mm × 3 mm pieces, which were plated in a 35-mm dish. Fibroblasts that migrated out of the tissue were subsequently trypsinized and expanded. 1014 EF was used as a control cell line for subsequent differentiation experiments and cell surface marker analysis.

Unless otherwise noted, cells were cultured in expansion medium: high glucose DMEM (Gibco Life Technologies 12100–046) with 10% fetal bovine serum (FBS, JR Scientific), in 5% CO_2_ at 37°C. Cells were expanded by passaging at 1:3 ratio at each passage, and were cryopreserved in 75% DMEM, 10% DMSO and 15% FBS, to be thawed at corresponding passages for subsequent experiments. Passage 5 (P5) cells were used for all experiments comparing different MSC lines with fibroblasts. 9004 BM-MSC at passage 10 (P10), passage 15 (P15) and passage 20 (P20) cells were used for all experiments comparing MSCs of different passages. MSCs and fibroblasts were counted with a hemocytometer and seeded the same way within each experiment. The term ‘MSCs’ in this report will be used to refer to both BM-MSCs and ASCs.

### Colony-forming unit assay

MSCs were counted and 150 cells were seeded in a 10-cm dish. The cells were then cultured in 10 mL expansion medium for 10 d, without physical disturbance or medium changes. At Day 10, cells were rinsed with phosphate-buffered saline (PBS), fixed with 4% paraformaldehyde for 30 min, and stained with 0.5% crystal violet solution for 10 min to visualize colonies. The number of colonies per dish was determined under light microscopy, with colonies of 50 cells or more counted as positive. Colony-forming efficiency (CFE) was then expressed as the percentage of colonies over the number of cells plated at Day 0. This assay was done for each cell line in triplicates (P5 MSCs) or 4 replicates (P10-20 MSCs), and the experiment was repeated once more. Data from all replicates were combined to get the average percent GFP-positive cells for each cell line.

### Karyotype analysis

To harvest mitotic chromosomes for karyotype analysis, MSCs were cultured to 60-70% confluency and colcemid was added to the culture medium at 0.07 μg/mL. After incubation for 60 min at 37°C, culture medium was discarded and the cells were trypsinized and centrifuged. The cell pellet was resuspended in 0.075 mol/L KCl hypotonic salt solution and incubated at 37°C for 20 min. Then, Carnoy’s fixative (3 absolute methanol:1 glacial acetic acid) was added to the suspension, after which cells were centrifuged and the supernatant discarded. The cell pellet was resuspended in Carnoy’s fixative and incubated at room temperature for 15 min, centrifuged again and the fixative wash procedure repeated twice. Finally, the cell pellet was resuspended in fixative and the cell suspension dropped from a Pasteur pipette onto cold, wet slides. Slides were air dried overnight, stained with 3% Giemsa solution and air dried. Metaphase spreads were visualized under light microscopy at 100X magnification, and images were taken using an attached camera. Chromosomes were then counted using ImageJ (National Institutes of Health) to determine total chromosome number and presence or absence of chromosomal abnormalities. Non-overlapping chromosome spreads were picked for counting that were roughly circular, with chromosomes spread sufficiently to enable counting. For each cell line, at least 20 spreads were counted.

### Osteogenic differentiation assay

MSCs and fibroblasts were seeded at a density of 10,000 cells/cm^2^ in 6-well plates and cultured in expansion medium to approximately 80% confluency in 2 d, then the medium was replaced by osteogenic differentiation medium: DMEM, 10% FBS, 100 nmol/L dexamethasone (Sigma), 0.05 mmol/L ascorbic acid-2-phosphate (Sigma) and 10 mmol/L β-glycerophosphate (Sigma). Cells were cultured in differentiation medium for 21 d, with medium changes every 3 d. Control cells were cultured in expansion medium in the same 6-well plate. After 21 d, cells were rinsed with PBS twice, fixed with 4% paraformaldehyde for 30 min, rinsed three times with distilled water, covered with 2% Alizarin Red S solution and incubated at room temperature for 10 min. Cells were then rinsed with distilled water and visualized under light and phase microscopy.

### Chondrogenic differentiation assay

Chondrogenic differentiation was carried out using the micromass method described by Zuk et al. [4]. MSCs and fibroblasts were trypsinized and resuspended in expansion medium to a concentration of 1 × 10^7^ cells/mL. Then, 10 μL drops of approximately 1 × 10^5^ cells of the cell suspension was carefully pipetted into the wells of 6-well plates. The cells were incubated for 2 h to allow for attachment, and then the wells were carefully filled with differentiation medium (or expansion medium for control cells) without disturbing the cell clumps. Chondrogenic differentiation medium consisted of DMEM, 10% FBS, 0.1 μmol/L dexamethasone, 50 μg/mL ascorbic acid-2-phosphate, 1X Insulin-Transferrin-Selenium (Life Technologies), and 10 ng/mL TGF-β3 (Life Technologies), which was added fresh each time. Cells were cultured for 21 d, with medium changes every 3 d. Cells were rinsed with PBS and fixed with 4% paraformaldehyde at room temperature for 15 min, after which they were incubated in 0.1 N HCl (pH 1) for 5 min, then stained with 1% Alcian Blue in 0.1 N HCl overnight. Cells were rinsed twice with 0.1 N HCl for 5 min with shaking to remove non-specific staining, after which they were air-dried and visualized under light microscopy.

### Adipogenic differentiation assay

Two methods of inducing adipogenic differentiation were investigated. In the first method (designated “Adipo I”), MSCs and fibroblasts were plated in 6-well plates at a density of 10,000 cells/cm^2^ and cultured to 80% confluency. Then, expansion medium was replaced with adipogenic induction medium: DMEM, 10% FBS, 1.0 μmol/L dexamethasone, 0.5 mmol/L 3-isobutyl-1-methylxanthine (Sigma), 200 μmol/L indomethacin (Sigma) and 10 μmol/L insulin (Akron Biotech). Cells were cultured for 21 d with medium changes every 3 d.

In the second method (designated “Adipo II”), adipogenesis was induced using the method described by Pittenger et al. [34], with modifications. MSCs and fibroblasts were plated in 6-well plates at a density of 10,000 cells/cm^2^ and cultured to full confluency. Then, expansion medium was replaced with adipogenic induction medium (as described above) for 3 d, after which the medium was replaced with adipogenic maintenance medium – DMEM, 10% FBS, and 10 μmol/L insulin – for 1 d, followed by a return to adipogenic induction medium. After three cycles of induction, cells where maintained in adipogenic maintenance medium for another 7 d.

In both methods, control cells were cultured in expansion medium instead of induction medium for the length of the experiments. At the end of the assays, cells were rinsed with PBS twice and fixed with 4% paraformaldehyde for 60 min. Cells were then rinsed with distilled water and incubated in 60% isopropanol for 2–5 min. Finally, cells were covered with Oil Red O solution for 5 min, rinsed, and covered with hematoxylin as a counter-stain for 1 min. Stained cells were visualized under light microscopy. To quantify adipogenic differentiation capacity, images of cells were captured to represent at least 4,000 cells per replicate of each cell line. Lipid-positive cells that stained with Oil Red O were counted using ImageJ (National Institutes of Health). When lipid-positive cells were too sparse, each well of the plate was divided into sectors, and positive cells were counted by hand using the microscope. Adipocyte count is expressed as the percentage of lipid-positive cells over the total number of nuclei counted. This assay was done in triplicates and the experiment repeated once more.

### RNA extraction and cDNA synthesis

Prior to RNA extraction, cells were harvested, centrifuged and flash frozen as pellets. For measuring the expression of cell surface markers, cells were harvested at 70-80% confluency. For measuring expression of adipogenic genes, cells were harvested at the end of the differentiation assay. Total RNA was extracted using TRIzol Reagent (Life Technologies) according to the manufacturer’s instructions. RNA quality was confirmed via agarose gel electrophoresis, and 1 μg of total RNA was subsequently DNAse-treated (Thermo Scientific) and used for first-strand cDNA synthesis using Oligo(dT) primers and RevertAid First Strand cDNA Synthesis Kit (Thermo Scientific) according to the manufacturer’s instructions.

### RT-PCR

Published primer sequences were used for amplifying *GAPDH*, *OCT4*, *SOX2*[35] and *NANOG*[36]. Primer sequences for amplifying *THY1* (CD90), *NT5E* (CD73), *ENG* (CD105), *PTPRC* (CD45) and *CD34* (CD34) were designed based on caprine or bovine sequences using Primer3 and spanned exon junctions or included introns, to control for possible genomic DNA contamination [Table 1]. Cycling parameters for amplifying *GAPDH*, *OCT4*, *SOX2* and *NANOG* were acquired from the papers referenced, whereas cycling parameters used for amplifying the other genes were as follows: initial denaturation at 94°C for 2 min, followed by 32 cycles of denaturation at 94°C for 30 s, annealing at respective temperatures for 30 s, 72°C for 40 s, and then a final extension at 72°C for 5 min.

**Table 1:** **Primer sequences used in RT-PCR**

Gene	Primer sequences (5’ → 3’)	Product size, bp	Anneal T, °C	Source
*GAPDH*	F: TCATGACCACAGTCCATGCCATCACT	253	60	[35]
	R: GATGTCATCATATTTGGCAGGTTTCTCC			
*OCT4*	F: AGGTGTTCAGCCAAACGACTATCTG	192	55	[35]
	R: TCGGTTCTCGATACTTGTCCGCTT			
*NANOG*	F: GCCGAGGAATAGCAATGG	444	53	F: AY786437.2
	R: TACAAATCTTCAGGCTGTATGTTG			R: [36]
*SOX2*	F: TGCAGTACAACTCCATGACCAGCT	281	55	[35]
	R: GTAGTGCTGGGACATGTGAAGTCTG			
CD90	F: GCACCATGAACCCTACCATC	241	55	NM_001034765.1
(*THY1*)	R: TTGGTTCGGGAGCTGTATTC			
CD73	F: CTGAGACACCCGGATGAGAT	160	57	NM_174129.3
(*NT5E*)	R: ACTGGACCAGGTCAAAGGTG			
CD105	F: AGATGCCAACATCACACAGC	129	60	NM_001076397.1
(*ENG*)	R: TCCAGACGAAGGAAGATGCT			
CD45	F: GGGAGGAGGGAAAGCAAACC	146	60	XM_005691061.1
(*PTPRC*)	R: GCAGCTCTTCCCCATTCCAG			
CD34	F: AGGTGTGCTCCTTGCTCCT	134	55	NM_174009
(*CD34*)	R: GCCCATCTCTCTCAGGTCAG			

The sequences of primers used are listed with the genes amplified, PCR product sizes (bp), annealing temperatures and sources. Primers were acquired from the indicated references, or designed using Primer3 based on the GenBank accessions provided.

### Quantitative RT-PCR

Primers for amplifying *GAPDH*[37], CD90, CD105, CD73, *PPARG*[38] and *FABP4*[39] are listed in Table 2. Primer sets that were not obtained from references were designed based on caprine or bovine sequences using Primer3 and spanned exon junctions or included introns. mRNA expression of these genes was quantified using Fast SYBR Green Master Mix (Life Technologies) on the ABI 7500 Fast thermocycler (Applied Biosystems), using the following thermocycling parameters: 95°C for 20 s, followed by 40 cycles of 95°C for 3 s and 60°C for 30 s, followed by a melt curve stage of 95°C for 15 s, 60°C for 1 minute, 95°C for 15 s and 60°C for 15 s. Relative gene expression was obtained by normalizing with GAPDH expression as the internal control and using the 2^-ΔΔCT^ method [40, 41], calculating differences in mRNA expression as fold changes relative to expression in 1014 EF for comparisons between cell lines, or to 9004 BM-MSC at P5 for comparisons between passages.

**Table 2:** **Primer sequences used in quantitative RT-PCR**

Gene	Primer sequences (5’ → 3’)	Product size, bp	Anneal T, °C	Source
*GAPDH*	F: TTGTGATGGGCGTGAACC	127	60	[37]
	R: CCCTCCACGATGCCAAA			
*PPARG*	F: AAAGCGTCAGGGTTCCACTA	201	60	[38]
	R: CCCGAACCTGATGGCGTTAT			
*FABP4*	F: TGAGATGTCCTTCAAATTGGG	101	60	[39]
	R: CTTGTACCAGAGCACCTTCATC			
CD90	F: GAATCCCACCGTCTCCAATA	190	60	XM_005689553.1
(*THY1*)	R: CTTGTGGCTTCCTGTGTCCT			
CD73	F: CTGAGACACCCGGATGAGAT	160	57	NM_174129.3
(*NT5E*)	R: ACTGGACCAGGTCAAAGGTG			
CD105	F: GCAAAGCATCTTCCTTCGTC	56	60	NM_001076397.1
(*ENG*)	R: GATTGCAGGAGAACGGTGAG			

The sequences of primers used are listed with the genes amplified, PCR product sizes (bp), annealing temperatures and sources. Primers were acquired from the indicated references, or designed using Primer3 based on the GenBank accessions provided.

### Immunofluorescence

Cells of each cell line were plated into a 4-well plate. At 50-70% confluency after 2 d, the cells were rinsed with PBS twice and fixed with 4% paraformaldehyde for 10 min at room temperature. After rinsing with PBS three times, the cells were blocked with PBS-Tween (PBST) and 3% normal donkey serum (NDS) for 30 min at room temperature. Cells were probed with a goat polyclonal primary antibody against human OCT4 (Santa Cruz Biotechnology) (1:300) in PBST and 1% NDS overnight at 4°C with shaking. After that, the cells were rinsed with PBST and 1% NDS three times for 10 min each, and then probed with a donkey anti-goat secondary antibody (1:500), conjugated with Alexafluor 488, in PBST and 1% NDS for 1 h at room temperature with shaking. Then, the cells were rinsed in PBST three times for 10 min each. In the last rinse, Hoescht stain was added at 10 μg/mL to stain the nuclei. The wells were kept wet in PBS and the cells were visualized under fluorescent microscopy. Bovine blastocysts were used as a positive control. Blastocysts were rinsed, blocked and probed as described above, then stored in PBST until visualization. For visualization, stained blastocysts were mounted on a glass slide in Prolong Gold Anti-Fade reagent (Life Technologies).

### Transfection efficiency of circular GFP (transient)

For transfection experiments, plasmid pEGFP-N1 (Clontech) was used. This 4.7 kb plasmid contains the enhanced green fluorescent protein gene (EGFP) controlled by a human cytomegalovirus (CMV) promoter and the neomycin resistance gene (Neo^R^) controlled by a simian virus-40 (SV40) promoter. Plasmid DNA was isolated using PerfectPrep EndoFree Plasmid Maxi Kit (5 Prime) according to manufacturer’s instructions, and then concentrated using a vacuum centrifuge until DNA concentration was at least 1 μg/μL for use in transfection.

MSCs were counted with a hemocytometer, and 500,000 cells were aliquoted for transfection with 10 μg plasmid DNA. Transfection was performed via the Nucleofector II system (Lonza, Amaxa, Germany), using the Human MSC Nucleofector kit. The bone marrow-derived cell lines (9004 BM-MSC and 9003 BM-MSC) were Nucleofected using program G-017, whereas the adipose-derived cell line (9003 ASC) were Nucleofected using program C-020. After transfection, cells were plated in a T-25 flask for culture in expansion medium. Fluorescent cells were counted at 24 h post-transfection using a fluorescent microscope. Transfection efficiency was expressed as the percentage of green cells over total cells counted. This assay was done for each cell line in triplicates (P5 MSCs) or 4 replicates (P10-20 MSCs), and the experiment was repeated once more. Data from all replicates were combined to get the average percent colonies for each cell line.

### Integration of Neo^R^ construct

For these transfection experiments, pEGFP-N1 was isolated using PerfectPrep EndoFree Plasmid Maxi Kit (5 Prime) according to manufacturer’s instructions, and then linearized by ApaLI digestion and purified by ethanol precipitation. Plasmid DNA was resuspended in UltraPure H_2_O (Gibco Life Technologies) such that DNA concentration was at least 1 μg/μL for use in transfection. MSCs were counted with a hemocytometer, and 500,000 cells were aliquoted for transfection with 10 μg linearized plasmid DNA as described in the previous section. After transfection, cells were plated in a T-25 flask for culture in expansion medium. At 48 h post-transfection, expansion medium was aspirated and replaced with selective medium: DMEM, 10% FBS and 800 μg/mL G418 (Gibco Life Technologies). Bone marrow-derived MSCs (9004 BM-MSC and 9003 BM-MSC) were maintained in selection for 12 d, whereas adipose-derived MSCs (9003 ASC) required a longer selection window of 16 d. At the end of selection, cells were rinsed with PBS and fixed with 4% paraformaldehyde for 30 min. Then, cells were stained with 0.5% crystal violet solution for 10 min to visualize neomycin-resistant colonies. Only discrete colonies that measured ≥ 2 mm in diameter were counted. Integration of linear pEGFP-N1 was measured by taking the number of integrant colonies per total cells transfected. This assay was performed for each cell line in 4 replicates and the experiment repeated once more. Data from all 8 replicates were combined to get the average number of integrant colonies for each cell line.

### Statistical analysis

Using the software SAS 9.3 (SAS Institute), data was analyzed using one-way ANOVA and statistically grouped using Tukey’s multiple means comparison test. Data was blocked by experiment, or the blocks removed when the block effect was not significant. *p* values are reported for the ANOVA, while statistical differences with a *p* value of less than 0.05 was considered as significant for the Tukey analysis. Data is represented as means ± SEM.

## Results

### Colony-forming efficiency (CFE)

**Figure 1:**
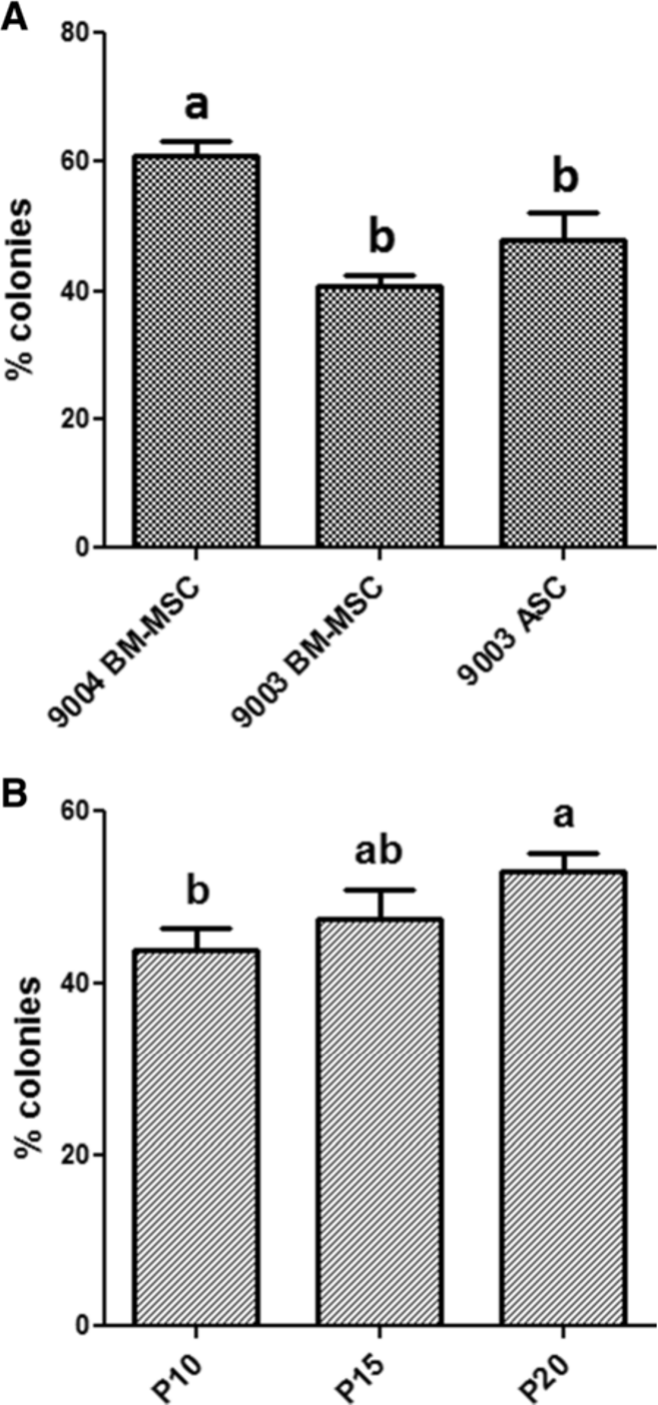
**Colony-forming efficiency of MSCs.** Colony-forming efficiency (CFE) of P5 MSCs from different sources **(A)** and 9004 BM-MSC cultured to passages 10, 15 and 20 **(B)**. CFE is expressed as percentage of colonies formed per total cells plated and presented as means (± SEM). Statistically significant groups by Tukey’s test are indicated by letters (*P* < 0.05).

All three MSC lines were able to form colonies after low-density plating (150 cells/55 cm^2^). As MSCs are heterogeneous, colonies formed were a mixture of dark, dense colonies of smaller, proliferative cells [Additional file 1: Figure S1A] and colonies of larger, flatter cells that were lighter in color [Additional file 1: Figure S1B]. At P5, there were significant differences in CFE between the cell lines (*P* = 0.0008). 9004 BM-MSC exhibited a CFE of 61.0% ± 2.2, which was significantly higher (*P* < 0.05) than those of 9003 ASC (48.0 ± 4.2) and 9003 BM-MSC (40.7 ± 1.9) [Figure 1A].

To investigate the effect of passaging on CFE and other MSC characteristics, 9004 BM-MSC was expanded to passages 10, 15 and 20. At higher passages, 9004 BM-MSC retained colony-forming ability with statistical differences observed in CFE between passages (*P* = 0.03). The highest CFE was observed in P20 MSCs, in which 53.0% ± 2.1 of cells plated formed colonies. P15 MSCs exhibited a CFE of 47.4% ± 3.4, while P10 MSCs yielded a CFE of 43.8% ± 2.7, which was significantly lower compared to P20 MSCs (*P* < 0.05) [Figure 1B].

### Karyotype analysis

All three MSC lines demonstrated chromosomal stability *in vitro*, at least up to 5 passages in culture. For each cell line, the majority of chromosome spreads showed a normal ploidy level of 2N = 60, with no sign of gross chromosomal abnormalities observed. The incomplete spreads were usually characterized by the loss of 1 to 3 chromosomes, which is consistent with chromosome loss caused by the spreading technique. Examination of complete metaphase spreads isolated from 9004 BM-MSC at P10, P15 and P20 revealed a complete set of 60 chromosomes with no sign of gross chromosomal abnormalities.

### Osteogenic differentiation

**Figure 2:**
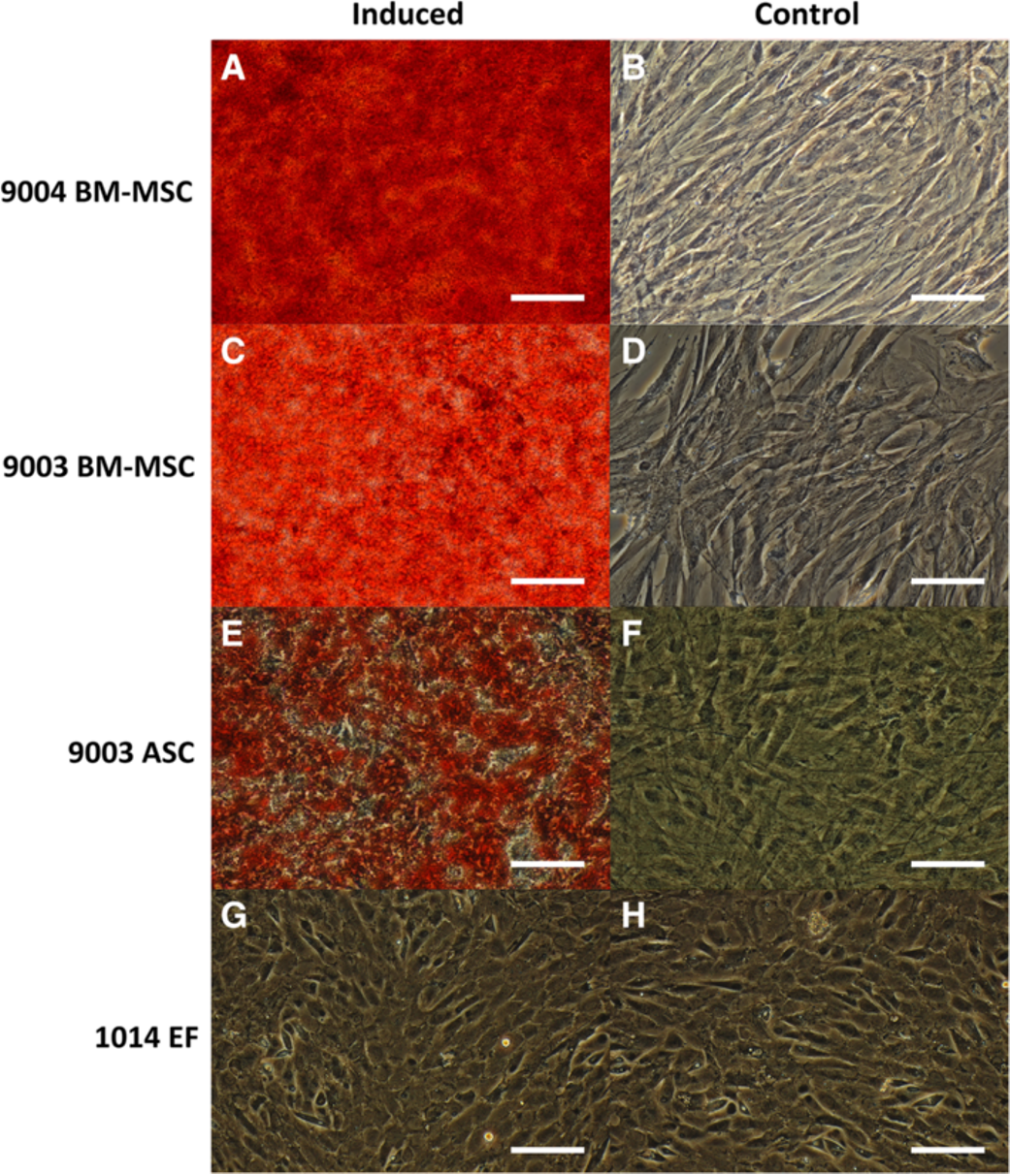
**Osteogenic differentiation of MSCs and fibroblasts.** P5 MSCs from 9004 BM-MSC **(A, B)**, 9003 BM-MSC **(C, D)**, 9003 ASC **(E, F)** and 1014 EF **(G, H)** were cultured in osteogenic differentiation or control medium, respectively and stained with Alizarin Red S, which stains calcium deposits red. Calcium mineralization in 9003 ASC **(E)** appeared less extensive than that of the BM-MSCs **(A and C)**. 1014 EF failed to undergo osteogenesis, as evident from the absence of staining. **(G, H;)**. Representative images are shown at 200X magnification. Scale bars represent 100 μm.

All three MSC lines showed capacity for undergoing induced osteogenic differentiation [Figure 2]. Mineralized calcium deposits stained orange-red with Alizarin Red S. Control MSCs and the fibroblast line did not exhibit calcium deposition, showing inability to undergo induced osteogenic differentiation. Differences in the extent of calcium mineralization between the MSC lines can be observed by visual comparison. The bone-marrow derived MSCs lines appear to have higher capacity for osteogenic differentiation, as shown by the greater extent of staining in 9004 BM-MSC [Figure 2A] and 9003 BM-MSC [Figure 2C], compared to the adipose-derived 9003 ASC [Figure 2E].

### Chondrogenic differentiation

**Figure 3:**
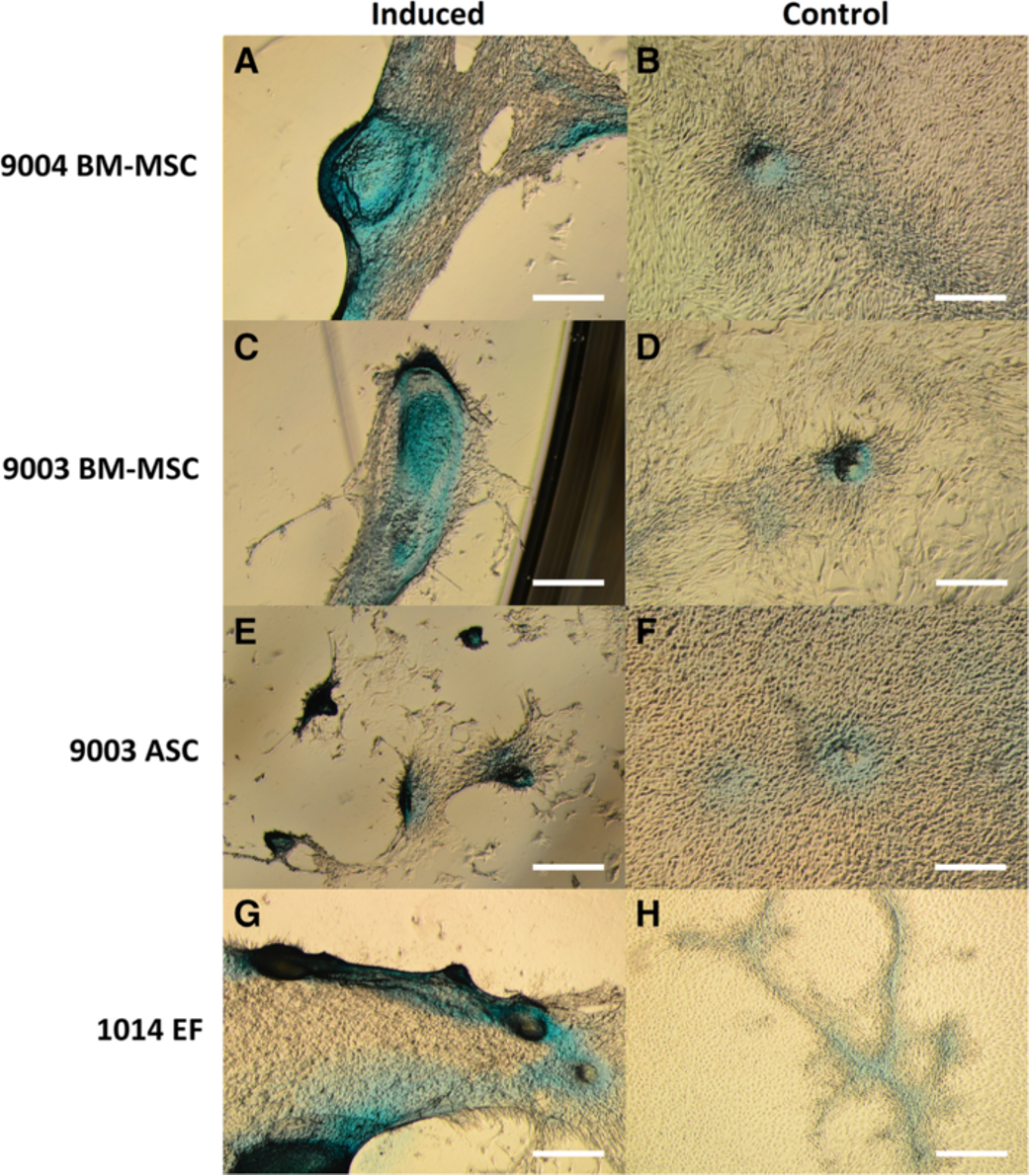
**Chondrogenic differentiation of MSCs and fibroblasts.** P5 MSCs from 9004 BM-MSC **(A, B)**, 9003 BM-MSC **(C, D)**, 9003 ASC **(E, F)** and 1014 EF **(G, H)** were cultured in chondrogenic differentiation or control medium, respectively and stained with Alcian Blue, which stains cartilage blue. Cellular condensation, as well as ridge and micromass formations that stain positive were observed in 9004 BM-MSC, 9003 BM-MSC, 9003 ASC and 1014 EF cultured in chondrogenic medium (**A**, **C**, **E**, **G** respectively). Some staining was observed in cells cultured in control medium, but cells generally remained in monolayer (**B**, **D**, **F**, **H** respectively). Representative images are shown in phase contrast at 40X magnification. Scale bars represent 500 μm.

MSCs and ear fibroblasts were able to undergo chondrogenesis. Differentiated cells underwent morphological changes, migrating to each other to form ridges and clumps, whereas control cells remained in monolayer [Figure 3]. These ridges and clumps of cells stained positive with Alcian Blue, which stains sulfated glycosaminoglycans prevalent in cartilage. Undifferentiated monolayers showed little to no staining. Fewer but larger micromasses were observed in the BM-MSCs, whereas the ASCs yielded numerous but smaller micromasses. In 1014 ear fibroblasts, there were large micromasses as well as small ones akin to those found in 9003 ASC.

### Adipogenic differentiation: cytochemical staining of adipocytes

**Figure 4:**
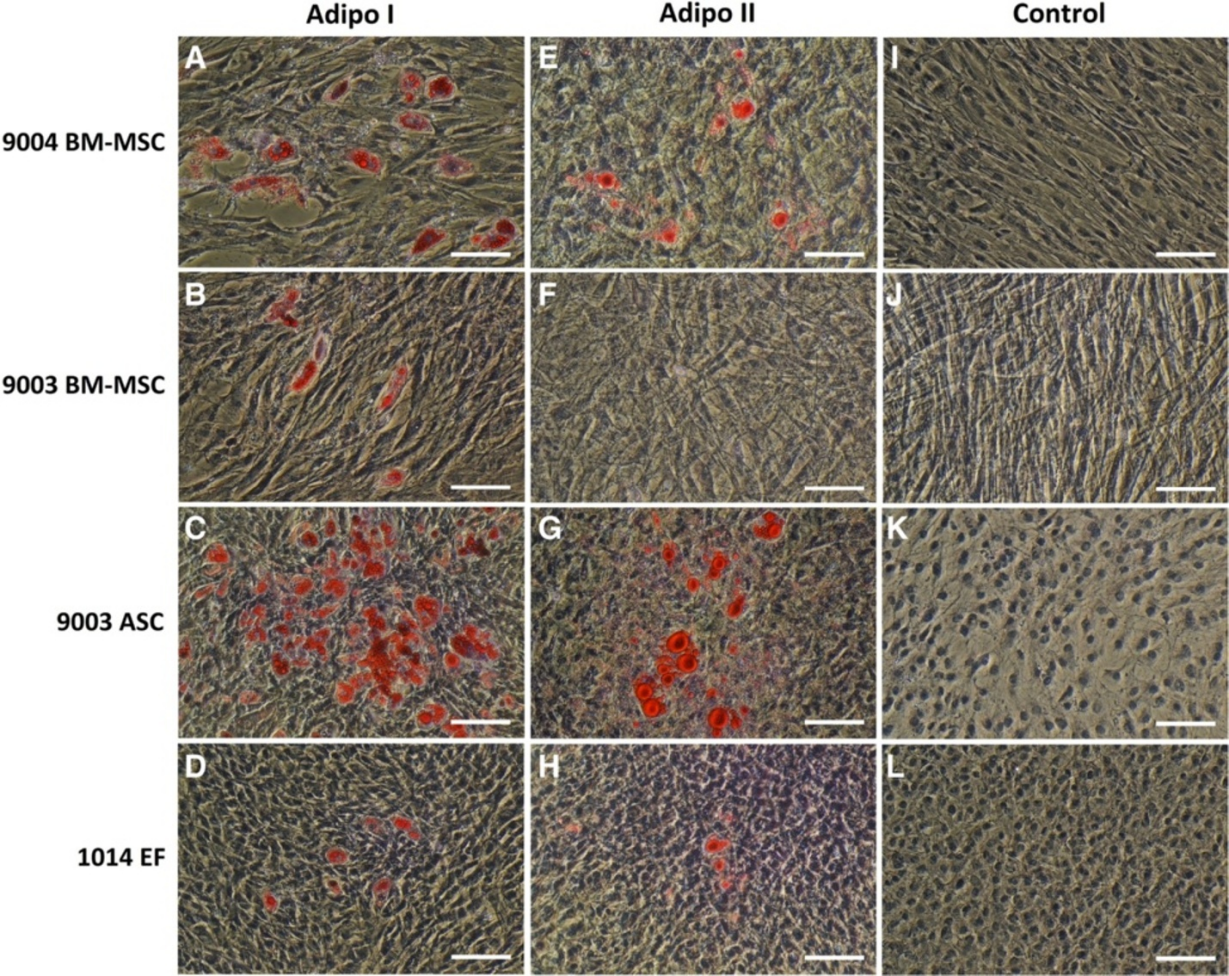
**Oil Red O staining of MSCs and fibroblasts differentiated using two methods of adipogenic induction.** P5 9004 BM-MSC **(A, E, I)**, 9003 BM-MSC **(B, F, J)**, 9003 ASC **(C, G, K)** and 1014 EF **(D, H, L)** were differentiated by the Adipo I method **(A-D)**, Adipo II method **(E-H)**, or cultured in expansion medium **(I-L)**. Differentiated adipocytes accumulated lipid droplets in the cytoplasm that stain red with Oil Red O. Cells cultured in control medium **(I-L)** and 9003 BM-MSC induced by Adipo II **(F)** did not yield lipid-filled adipocytes. Representative images are shown in phase contrast at 200X magnification. Scale bars represent 100 μm.

Both differentiation methods demonstrated the capacity of MSCs and ear fibroblasts to undergo adipogenic differentiation [Figure 4]. Control cells that were cultured in expansion medium did not undergo adipogenesis and did not stain positive with Oil Red O [Figure 4I-L]. However, there were visual differences in the morphology of adipocytes obtained from each differentiation method. Cells that were differentiated with the Adipo I method accumulated many small lipid droplets in the cytoplasm [Figure 4A-D], whereas there were visually fewer but bigger lipid droplets in cells differentiated using the Adipo II method [Figure 4E-H].

**Figure 5:**
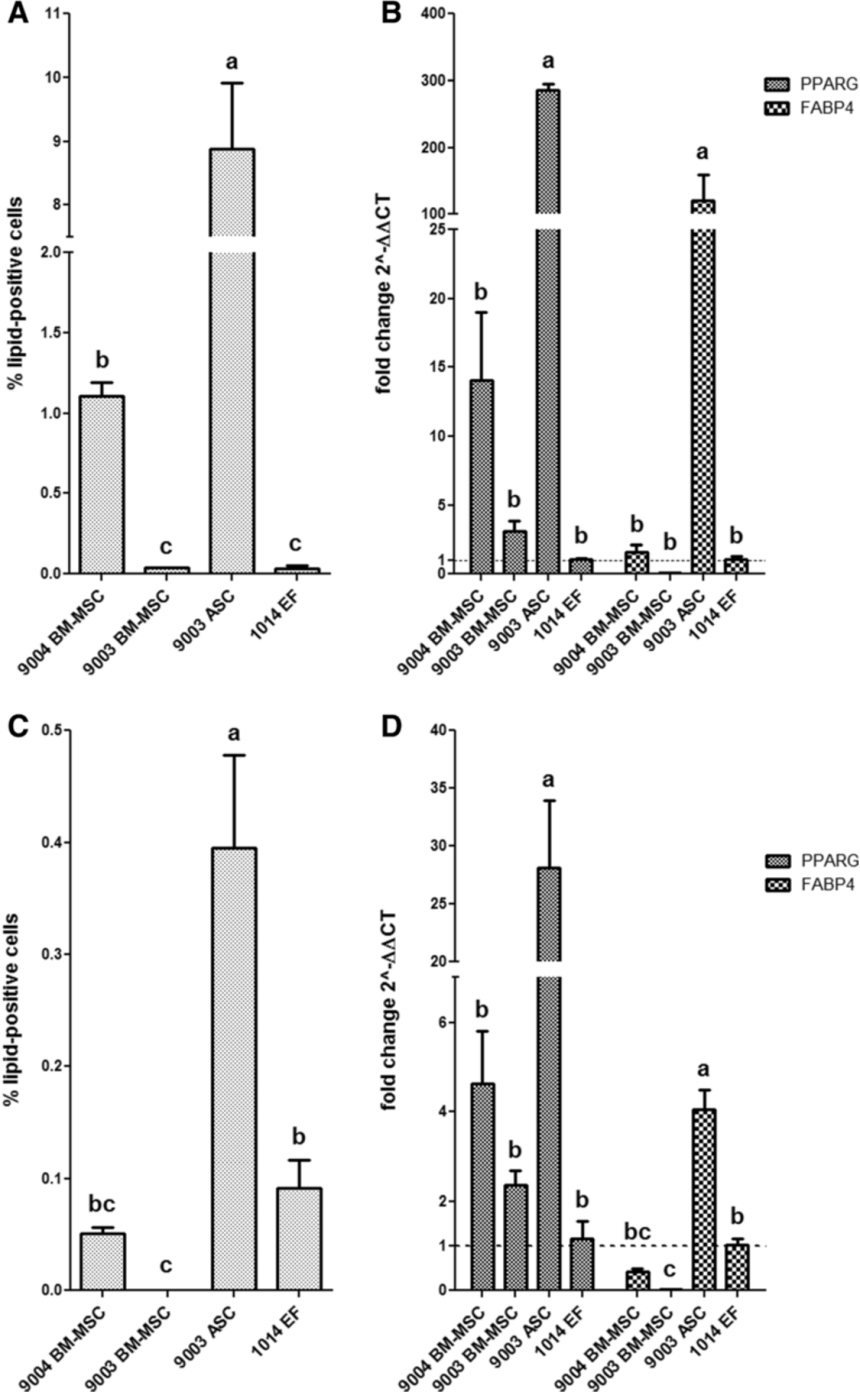
**Adipogenic differentiation capacity of P5 MSCs and fibroblasts.** The percentage of lipid-positive cells over total cells counted (± SEM) is shown for each cell line differentiated with Adipo I **(A)** and Adipo II **(C)**. Quantitative RT-PCR analysis of PPARG and FABP4 expression was performed on each cell line differentiated with Adipo I **(B)** and Adipo II **(D)**, and data is presented as fold change (± SEM) in expression relative to expression levels in 1014 EF (fold change ~1, indicated by the dotted line). Statistically significant groups by Tukey’s multiple means comparison test are indicated by letters (*P* < 0.05 for **A**-**D**).

Adipo I yielded greater percentages of lipid-positive cells, with significant differences between the cell lines (*P* < 0.0001) [Figure 5A]. 9003 ASC exhibited the highest percentage of lipid-positive cells (8.9% ± 1.0), significantly higher than the percentage observed in 9004 BM-MSC (1.1% ± 0.08, *P* < 0.05). 9003 BM-MSC and 1014 EF yielded the lowest percentage of lipid-positive cells (0.038% ± 0.001 and 0.036% ± 0.02, respectively), significantly lower than 9004 BM-MSC (*P* < 0.05). In Adipo II, the number of lipid-positive cells was much lower, but statistical differences were still found among the cell lines (*P* < 0.0001) [Figure 5C]. 9003 ASC still yielded the highest percentage of lipid positive cells (0.40% ± 0.08), significantly higher than 1014 EF (0.092% ± 0.02, *P* < 0.05). 9004 BM-MSC only produced 0.051% ± 0.005 lipid-filled cells, while 9003 BM-MSC induced with the Adipo II method did not yield any lipid-positive cells.

Upon visual observations throughout the 21-d assay period of Adipo I, it appeared that the numbers of adipocytes in 9004 BM-MSC and 9003 BM-MSC peaked at the midpoint of the assay, after which lipid-filled cells were lost. As the experiment was not planned to be a time-course, samples were not taken at this time point. Nevertheless, the BM-MSCs alone were differentiated with the Adipo I method for 10 d in a separate experiment and the results confirmed the earlier observations [Additional file 1: Figures S2 and S3]. As for 9003 ASC and 1014 EF, the number of adipocytes steadily increased throughout the 21-d period. This phenomenon was not observed in BM-MSCs differentiated in the Adipo II method, in which adipocyte numbers steadily increased without loss.

### Adipogenic differentiation: expression of adipogenesis marker genes

The differences in percentage of lipid-positive cells correlated with the differences seen in the mRNA expression of *PPARG* and *FABP4*. In Adipo I, there were significant differences in *PPARG* and *FABP4* expression among the cell lines (*P* < 0.0001 for both) [Figure 5B]. 9003 ASC showed the highest expression of *PPARG* at 286.4 ± 9.3-fold of expression in the control cell line 1014 EF, significantly higher than the other cell lines (*P* < 0.05). Expression of *PPARG* in 9004 BM-MSC and 9003 BM-MSC was higher than 1014 EF as well, but the difference was not significant. The expression of *FABP4* in 9003 ASC was also significantly higher than the other cell lines, at 120.7 ± 38.9-fold higher than the control (*P* < 0.05). The expression in 9004 BM-MSC was only 1.6 ± 0.6-fold higher than the control, whereas 9003 BM-MSC expressed fewer *FABP4* transcripts at 0.08 ± 0.01-fold of the control.

MSCs differentiated with the Adipo II method showed lower differential expression of these genes relative to the fibroblasts. Significant differences between the cell lines were observed for *PPARG* and *FABP4* (*P* < 0.0001 for both), and the highest expression of both genes was again seen in 9003 ASC [Figure 5D]. *PPARG* expression was significantly higher in 9003 ASC (28.2 ± 5.8-fold, *P* < 0.05) compared to the other cell lines, and consistently higher but not significantly so in the BM-MSCs relative to the fibroblasts, with 9004 BM-MSC showing 4.6 ± 1.2-fold and 9003 BM-MSC showing 2.4 ± 0.3-fold higher expression relative to 1014 EF. *FABP4* expression again was concordant with observations in adipocyte counts: 9003 ASC had significantly higher expression at 4.1 ± 0.4-fold above the control cell line 1014 EF (*P* < 0.05), while *FABP4* expression was lower in 9004 BM-MSC, at 0.42 ± 0.07-fold of the control. 9003 BM-MSC exhibited the lowest expression of these genes, even yielding significantly lower relative expression of *FABP4* than that of the fibroblasts (*P* < 0.05).

### Adipogenic differentiation during long-term culture

**Figure 6:**
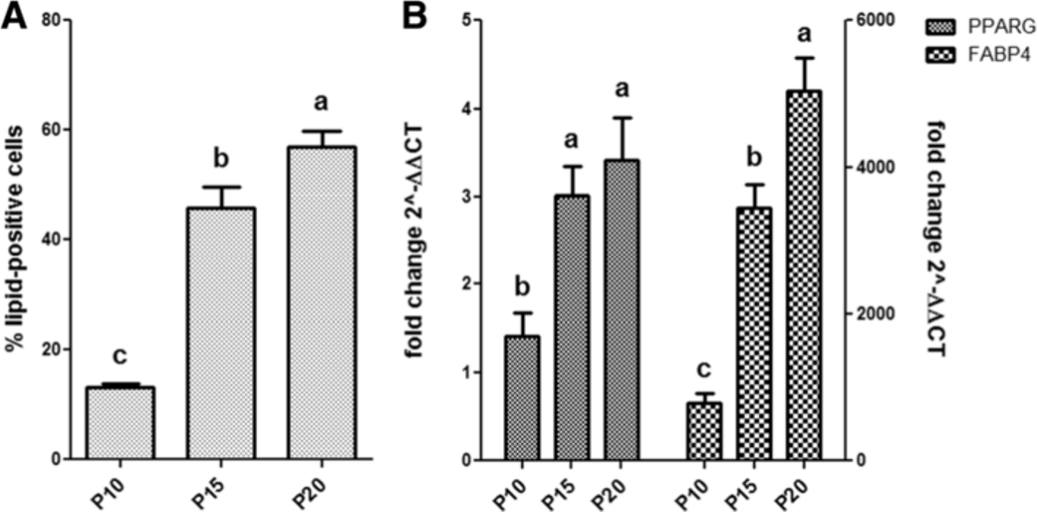
**Adipogenic differentiation capacity of BM-MSCs at higher passages.** The percentage of lipid-positive cells over total cells counted (± SEM) for 9004 BM-MSC at passages 10, 15 and 20 **(A)** and quantitative RT-PCR analysis of PPARG and FABP4 expression for each passage presented as fold change (± SEM) in expression relative to control cells at corresponding passages **(B)**. Statistically significant differences are indicated in letters according to analysis by Tukey’s multiple means comparison (*P* < 0.05).

The number of lipid-positive cells increased with increasing passages, and was significantly different between passages (*P* < 0.0001) [Figure 6A]. After 10 d of differentiation, 56.9% ± 2.9 of P20 MSCs stained positive for Oil Red O, which was significantly higher than the 45.9% ± 3.7 that stained positive in P15 MSCs (*P* < 0.05). P10 MSCs yielded 13.1% ± 0.7 lipid-positive cells, significantly lower than P15 and P20 MSCs (*P* < 0.05). The same trend was also observed in gene expression data, which is represented as relative to control, non-induced MSC cultures at the corresponding passage [Figure 6B]. Significant differences in *PPARG* and *FABP4* expression were observed as passage increased (*P* = 0.0006 and *P* < 0.0001, respectively). *PPARG* expression in P20 MSCs was the highest, at 3.4 ± 0.5-fold higher than the level in control non-induced P20 cells, followed by a 3.0 ± 0.3-fold increase in P15 MSCs. *PPARG* expression in P10 MSCs was significantly lower, at 1.4 ± 0.3-fold increase relative to control cultures (*P* < 0.05). In the case of *FABP4* expression, we observed the same pattern of significance as compared to the percentage of adipocytes counted. P20 MSCs expressed FABP4 at 5047.4 ± 434.8-fold higher relative to non-induced cells, significantly higher than expression in P15 cells at 3451.3 ± 320.0-fold (*P* < 0.05). *FABP4* expression was at 774.9 ± 136.0-fold relative to control cells in P10 cells, which was significantly lower compared to P15 and P20 cells (*P* < 0.05).

Upon further examination of the Oil Red O-stained cells, it was apparent that P15 and P20 MSCs contained more polygonal cells, compared to the largely spindle-shaped cells in P10 MSCs (data not shown). Control cells incubated in expansion medium did not yield lipid-positive cells (data not shown).

### Expression of cell surface marker genes

**Figure 7:**
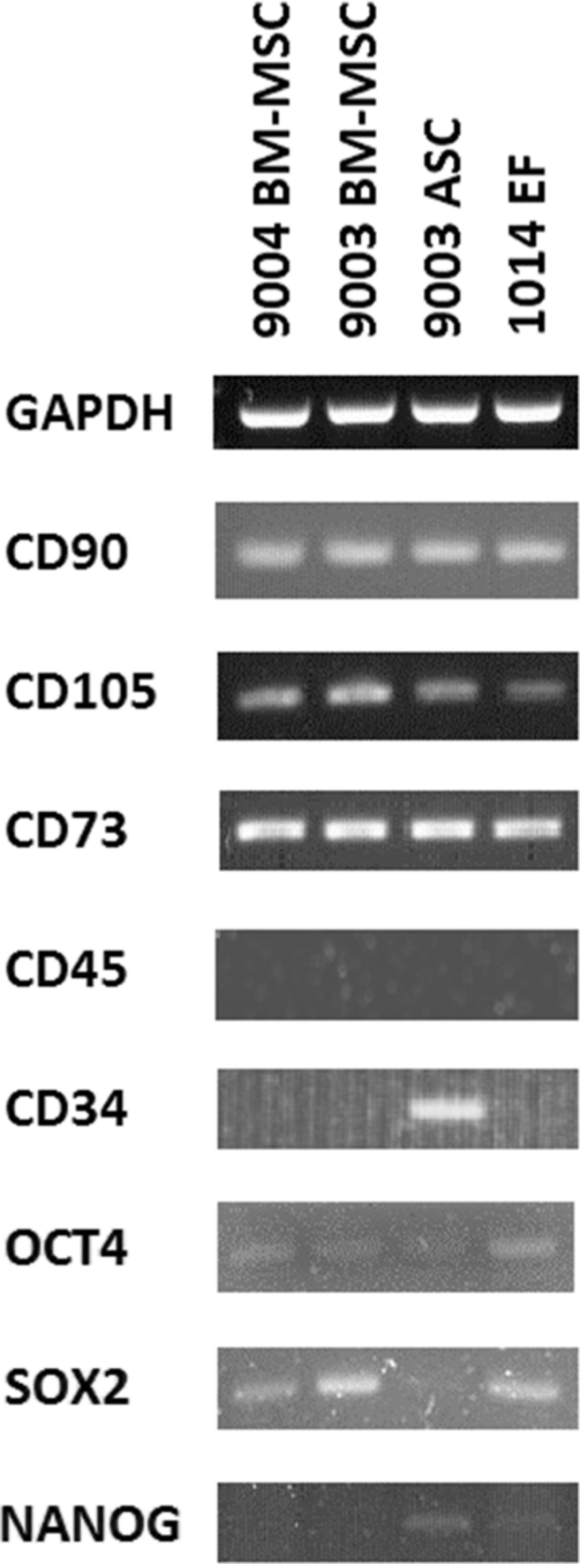
**RT-PCR analysis of gene expression in MSCs and fibroblasts.** RT-PCR was performed using primers that amplify the cell surface markers CD90, CD105, CD73, CD45 and CD34, and the pluripotency markers *OCT4*, *SOX2* and *NANOG*. Primers amplifying *GAPDH* were used as a positive control for the cDNA. PCR products were visualized in 2% agarose.

RT-PCR analysis revealed that all three MSC lines and 1014 EF expressed CD90, CD105 and CD73 [Figure 7], which are markers that have been defined as positive cell surface markers for human MSCs [5]. Expression of the hematopoietic marker CD45 was not detected in any of the cell lines, whereas CD34 was detected only in 9003 ASC [Figure 7].

**Figure 8:**
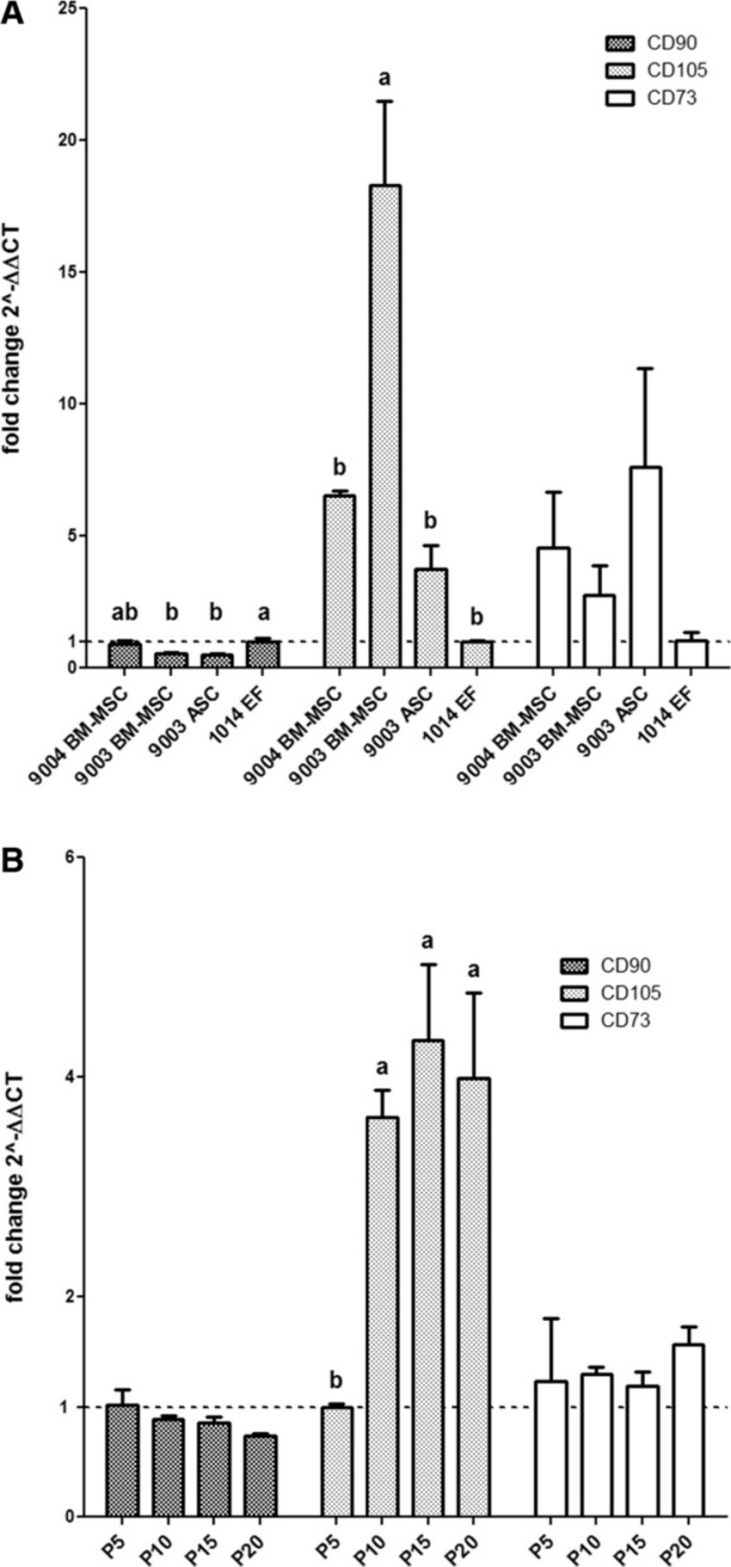
**Cell surface marker expression in MSCs and fibroblasts.** Quantitative RT-PCR was used to measure the expression of the cell surface markers CD90, CD105 and CD73 in P5 MSCs **(A)** and in 9004 BM-MSCs at passages 10, 15 and 20 **(B)**. Expression levels are presented as fold change (± SEM) relative to 1014 EF **(A)** or expression levels in P5 9004 BM-MSC **(B)** (fold change ~1, indicated by the dotted line). Statistically significant groups by Tukey’s multiple means comparison test are indicated by letters (*P* < 0.05). Significant differences between the cell lines were observed in CD90 and CD105 expression. Significant difference between the different passages was observed in CD105, but not in CD90 and CD73.

Quantitative RT-PCR analysis showed significant differences in CD90 expression (*P* = 0.0123), in that it was lower in MSCs compared to 1014 EF [Figure 8A]. CD90 expression in 9004 BM-MSC was 0.93 ± 0.1-fold of the expression in 1014 EF, whereas 9003-BM MSC and 9003 ASC showed significantly lower expression, with 0.54 ± 0.06-fold and 0.51 ± 0.02-fold expression compared to 1014 EF (*P* < 0.05). On the other hand, CD105 expression was overall higher in MSCs compared to 1014 EF (*P* = 0.0004), with 9003 BM-MSC showing significantly higher expression at 18.3 ± 3.2-fold compared to 1014 EF (*P* < 0.05). CD105 expression was 6.6 ± 0.2-fold and 3.8 ± 0.9-fold higher in 9004 BM-MSC and 9003 ASC, respectively, but the difference was not significant. CD73 expression was not significantly different in MSCs compared to 1014 EF (*P* = 0.2645).

To determine whether the expression of cell surface markers change during *ex vivo* expansion, quantitative PCR was performed on RNA isolated from 9004 BM-MSCs at P10, P15 and P20 to examine the gene expression of CD90, CD105 and CD73. Relative differences in mRNA expression were calculated using expression levels in P5 cells as the baseline. The expression of CD90 and CD73 were found to not change with passaging (*P* = 0.15 and *P* = 0.83 respectively) [Figure 8B]. However, the expression of CD105 increased significantly between P5 and P10 cells (*P* = 0.0084), with expression at 3.6 ± 0.2-fold in P10 MSCs relative to P5 MSCs (*P* < 0.05). CD105 expression appeared to plateau sometime after passage 10, showing no difference between P10, P15 and P20 MSCs.

### Expression of pluripotency genes and immunofluorescent detection of OCT4

RT-PCR showed that low expression of *OCT4* was detected in all three MSC lines as well as 1014 EF [Figure 7]. *SOX2* was also detected in the MSCs and fibroblasts, though expression in 9003 ASC appeared to be much lower. *NANOG* was faintly detected only in 9003 ASC and 1014 EF, and was either very lowly expressed or not expressed at all in 9004 BM-MSC and 9003 BM-MSC.

**Figure 9:**
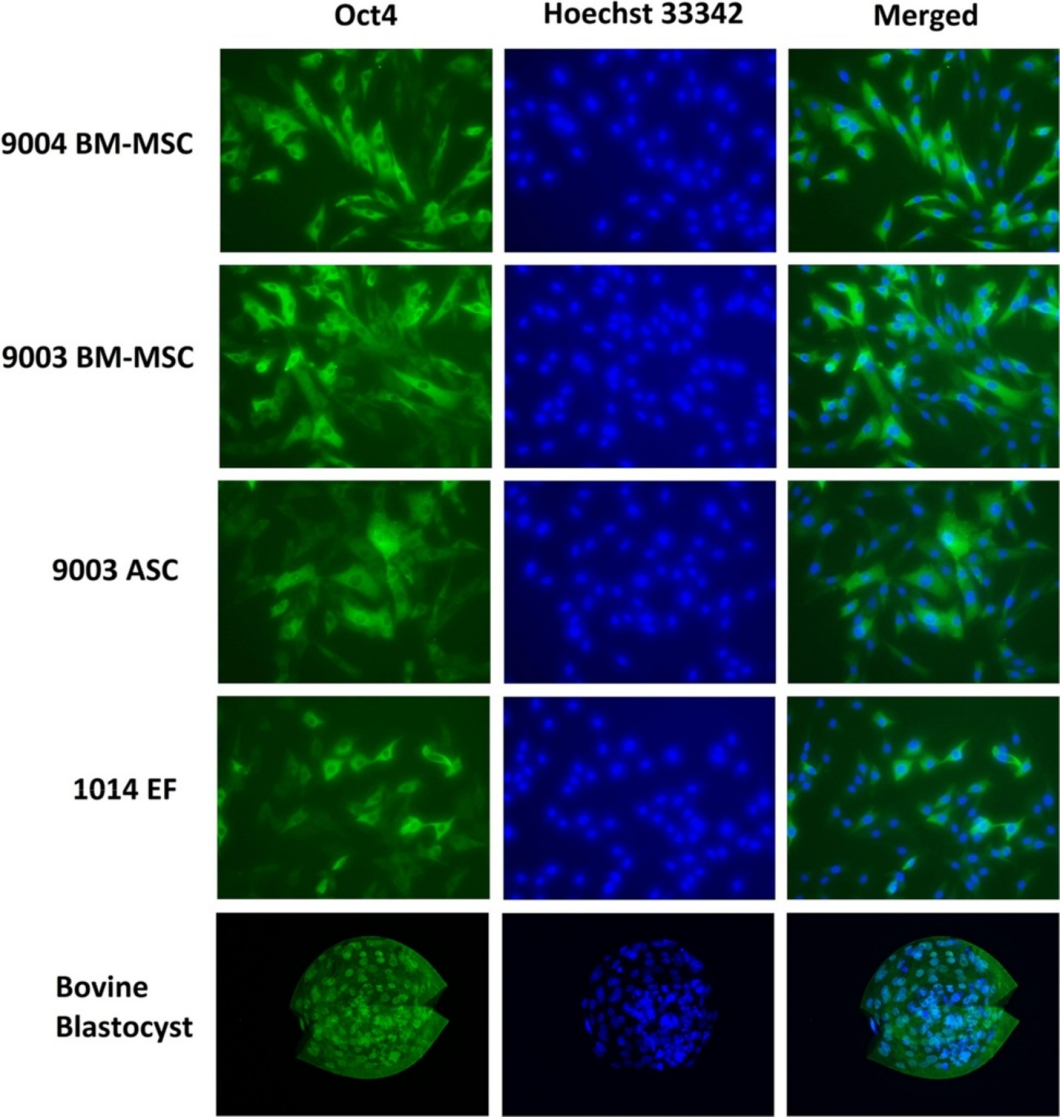
**Immunofluorescent staining of OCT4 in MSCs and fibroblasts.** P5 cells were stained with antibodies against OCT4. OCT4 was localized in the cytoplasm of MSCs and fibroblasts, whereas staining for OCT4 was specific to the nuclei in the bovine blastocyst. Representative images are shown at 200X magnification.

Immunofluorescent staining showed that all the MSC lines and the 1014 EF stained positive for OCT4 protein. Fluorescence was clearly restricted to the cytoplasm, while the nuclei remained dark and unstained [Figure 9]. Staining was also uneven – some cells fluoresced stronger than others, although it was not clear if this was a result of uneven OCT4 expression or due to uneven staining. Considering the faint bands detected from RT-PCR analysis, it is more likely that OCT4 expression in the cells was uneven. There seemed to be no difference in fluorescence intensity among the cell lines. In the bovine blastocysts, fluorescence was restricted to only the nuclei [Figure 9], consistent with known distribution of OCT4A protein in blastocysts [42, 43] and suggesting that the antibody used bound to both the OCT4A and OCT4B isoforms.

### Transfection and integration of introduced DNA

**Figure 10:**
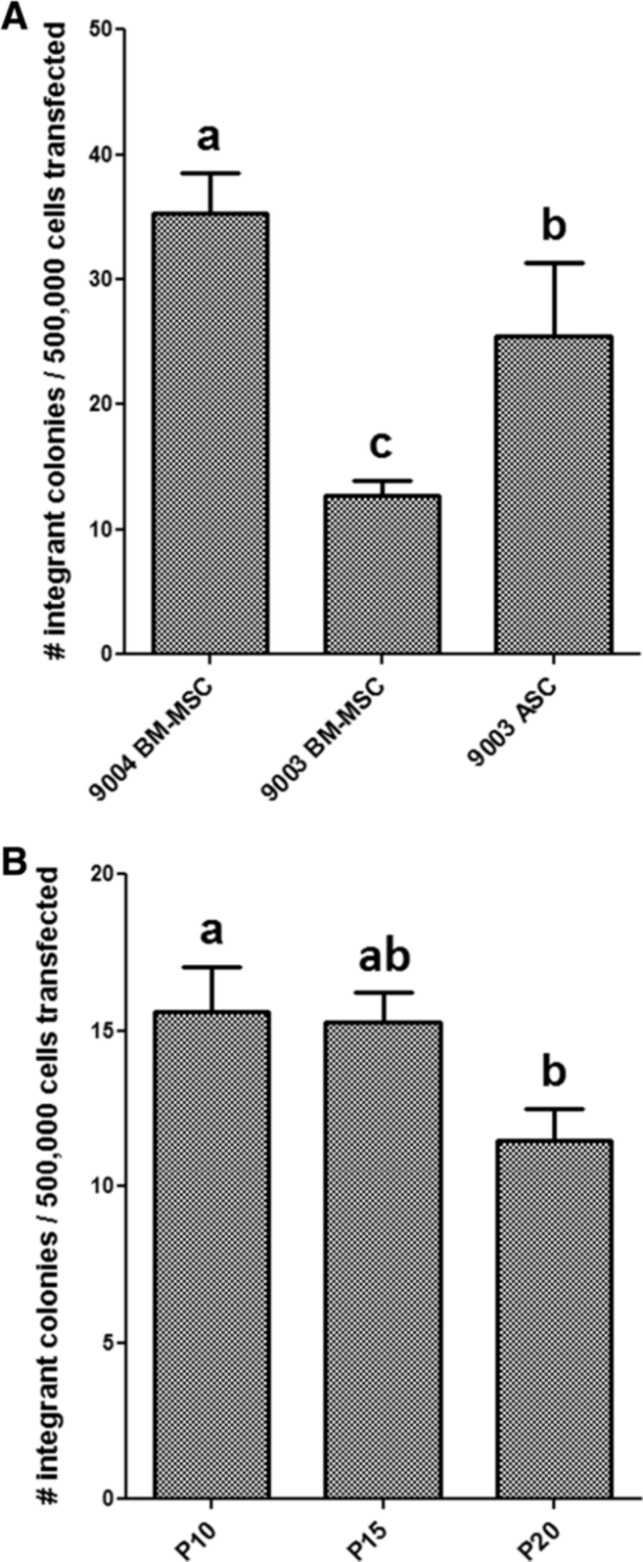
**Transfection and integration efficiency in MSCs.** P5 MSCs **(A)** and 9004 BM-MSC at passages 10, 15 and 20 **(B)** were transfected with pEGFP-N1. Integrant number were expressed as the number of discrete colonies that were resistant to G418 selection. Statistically significant groups by Tukey’s test is indicated by letters (*P* < 0.05). 9004 BM-MSC showed the highest number of integrants, followed by 9003 ASC and 9003 BM-MSC. 9004 BM-MSC showed a decreasing trend in number of integrants with increasing passages.

No significant difference was found in the percentage of GFP-positive cells among the MSC lines at P5 (*P* = 0.6049). Transfection efficiency was 45.3% ± 5.5 in 9004 BM-MSC, 50.4% ± 5.4 in 9003 BM-MSC and 51.7% ± 2.7 in 9003 ASC. Following transfection of linearized pEGFP-N1 and G418 selection in MSCs, surviving colonies of G418-resistant cells were counted. Significant differences in integration per 500,000 cells transfected were found (*P* < 0.0001). The number of integrant colonies was significantly different between each MSC line, with 9004 BM-MSC yielding the highest number of integrants (35.2 ± 3.3), followed by 9003 ASC (25.5 ± 5.8) and 9003 BM-MSC (12.8 ± 1.1) (*P* < 0.05) [Figure 10A].

To investigate whether long-term expansion of MSCs *in vitro* affect their potential for genetic modification, the transfection and integration of 9004 BM-MSCs at higher passages were examined. No significant difference was observed in the percentage of GFP-positive cells among MSCs at different passages (*P* = 0.62). Transfection efficiency was 24.4% ± 1.6 in P10 MSCs, 22.4% ± 1.5 in P15 MSCs and 23.4% ± 1.0 in P20 MSCs. Following transfection of linearized pEGFP-N1 and G418 selection in MSCs, a decreasing trend in integrant colony number was observed as passage number increased (*P* = 0.035). The number of integrants was the highest in P10 MSCs (15.6 ± 1.4), and progressively decreased in number in P15 MSCs (15.3 ± 1.0) and P20 MSCs (11.5 ± 1.0), with P20 having significantly fewer integrants than P10 (*P* < 0.05) [Figure 10B].

## Discussion

Our results indicate that the 9004 BM-MSC, 9003 BM-MSC and 9003 ASC are true mesenchymal stem cells, as evidenced by their ability to undergo trilineage differentiation [5]. 1014 EF was able to undergo adipogenic and chondrogenic differentiation, but not osteogenesis, hence distinguishing fibroblasts from MSCs. Fibroblasts can be isolated from multiple tissues; as a result, differentiation potentials reported for fibroblasts have been variable, such as being only capable of chondrogenesis [25, 44], exhibiting trilineage differentiation at a lower capacity [25, 45], or not at all [3]. In this study, goat ear fibroblasts lacked osteogenic capacity. In all cases, fibroblasts do not exhibit trilineage differentiation potential at the same capacity as MSCs, making it a more reliable method for distinguishing MSCs from fibroblasts, compared to cell surface marker expression.

Based on our results, adipogenic differentiation capacity in MSCs appears to be impacted by source tissue. Goat ASCs were more competent at adipogenesis than BM-MSCs, as reported for other species [8, 9]. This could be explained by intrinsic epigenetic differences that primes resident MSCs in different source tissues to differentiate into the surrounding tissue. This is consistent with the finding that MSCs derived from adipose, bone marrow and muscle have similar but not identical promoter methylation profiles [46]. According to a review by Boquest et al. [47], studies showed that undifferentiated ASCs are epigenetically primed for adipogenesis. We also saw significant differences between two lines of BM-MSCs isolated from two donor goats. Large donor-to-donor variability in adipogenic differentiation of BM-MSCs also was seen in a study by Aldridge et al. [48]. Even though observations on our goat MSCs are consistent with what has been seen in MSCs of other species, it is difficult to draw the same conclusions based on only one ASC line and two BM-MSC lines. Future studies should aim to solidify these findings with more MSC isolates.

In this study, fibroblasts derived from ear cartilage were observed to be capable of adipogenic differentiation at a low capacity, as seen with other isolated fibroblast lines [25, 48] and suggesting a rare subpopulation of mesenchymal progenitors reside in fibroblast preparations [49]. Consistent with our results, Alt et al. [25] also observed significantly more Oil Red O-staining adipocytes in human ASCs compared to skin fibroblasts. Stress-induced lipid accumulation in the fibroblasts is also a possibility, such as lipid formation due to overconfluence [50] and not adipogenesis.

After 21 d of adipogenic differentiation, the BM-MSCs appeared to exhibit a low adipogenic potential, and in some cases, even lower than the fibroblast cell line. However, from visual observations throughout the 21-d period of adipogenic differentiation via the continuous induction method (Adipo I), lipid-positive cells in BM-MSCs were seen to increase until approximately 10 d and appear to subsequently decrease in number. Adipogenic cell counts in both BM-MSC lines at Day 10 were found to be higher than the numbers acquired at Day 21 [Additional file 1: Figure S3]. Thus, the adipocyte count in BM-MSCs after 21 d of differentiation do not represent the maximal number that could be achieved in these cell lines, and although Adipo I did indeed yield greater numbers, this differentiation method was not able to sustain adipocyte numbers past two weeks of culture. The loss of adipocytes in BM-MSCs could either be explained by external aspects, such as composition of culture media, or intrinsic factors, such as the nature of the cells themselves. Bosch et al. [51] showed a plateau in number of lipid-positive cells after 10 d in porcine BM-MSCs. It is possible that the media compositions used are not sufficient to maintain BM MSCs differentiating down the adipogenic lineage, and needs to be further defined for less studied species such as goats and pigs.

In our study, we observed an increase in adipogenic differentiation capacity as passage number increased in 9004 BM-MSCs, as shown by adipocyte counts and mRNA expression of *PPARG* and *FABP4*. This is concordant with the observed increased adipogenesis in late-passage porcine BM-MSCs [52]. On the other hand, studies in rhesus macaque and human BM-MSCs show a decrease in adipogenic potential at higher passages [29, 30, 53]. These opposing results could be due to species-specific differences in the biology of MSCs. Also, an increase in CFE with passaging was observed, suggesting that the passage-related increase in adipogenic potential could also be attributed to the increased proportion of progenitor cells within the MSC culture. If this were the case, overall enhanced trilineage differentiation should be observed. Alternatively, should the increase in adipogenicity be due to shifts in the multipotentiality of the progenitor cells and not their numbers, an inverse effect between adipogenesis and osteogenesis is possible [54–57]. A concomitant increase in osteogenic potential has been demonstrated with the decrease in adipogenic potential [30, 53], or vice versa [52]. Further research should investigate the effects of passaging on the osteochondrogenic potential of goat BM-MSCs, and whether the same trend occurs in goat ASCs. This is especially relevant as goat MSCs are often utilized in bone and cartilage tissue engineering applications.

MSC cultures are known to be heterogeneous [58]. Therefore, each MSC isolation will have a heterogeneous mixture of cells – each may have a different proportion of mesenchymal progenitor cells, which is demonstrated by the difference in differentiation capacity and low density CFE shown in this work. The colony-forming unit (CFU) assay, or colony-forming efficiency assay, is an assay that measures the ability of a cell line or isolate to propagate and form colonies originating from single cells after plating at a very low density. CFU assays are useful in estimating the proportion of early progenitors in different preparations of MSCs [59]. A positive correlation between CFE and differentiation capacity have been demonstrated in other studies [29, 60, 61]. In this study, between the BM-MSC lines, the cell line with the higher CFE (9004 BM-MSC) was also observed to have higher differentiation capacity. When cultured through higher passages, 9004 BM-MSC exhibited an increase in CFE, suggesting that long-term *in vitro* culture, at least up to 20 passages, selects for progenitor cells as they can self-renew.

Goat MSC lines express CD90, CD105 and CD73, but do not express CD45, concordant with the minimal criteria set for defining human MSCs [5]. However, thus far, trilineage differentiation appears to be a more reliable method for identifying MSCs as it is consistent across species, whereas the expression of cell surface markers is more variable. For example, CD90 expression is variable in the mouse [62, 63] and is not expressed in rabbit MSCs [64]. Furthermore, the positive cell surface markers defined for MSCs are also expressed in fibroblasts [25, 31, 65], as shown in our study as well. Thus, these positive cell surface markers are not ideal for characterizing MSCs, given that MSCs share morphological and other characteristics with fibroblasts. Finding markers that distinguish MSCs from fibroblasts is important, as like MSCs [1], fibroblasts can also be found in virtually all tissues of the body and are morphologically similar to MSCs. As such, other research groups have endeavored to identify markers that distinguish MSCs from fibroblasts [31, 66], and also use fibroblasts as a control cell line in MSC characterization experiments [44, 67].

Data from qRT-PCR of cell surface markers showed that CD90 and CD73 mRNA levels did not change between passages 5 and 20. CD105 appeared to increase between passages 5 and 10, but did not change in further passages. CD105 was defined as a positive marker for its prevalence in MSCs at isolation [5], and was used to select for a more ‘pure’ MSC population [68]. Since no difference in CD105 expression was detected in 9004 BM-MSC between passages 10 and 20, while significant difference in adipogenesis was observed, it is unlikely that CD105 expression is related to adipogenic potential in MSCs. A study by Lysy et al. [69] also showed no link between CD105 expression and adipogenic capacity in MSCs and fibroblasts, whereas lung fibroblasts that also express CD105 were not able to undergo adipogenesis [25]. The fact that CFE in 9004 BM-MSC at passage 5 and 10 were comparable indicates that the increase in CD105 expression cannot be attributed to a growing number of progenitor cells. CD105, or endoglin, is a transmembrane protein involved in TGF-β signaling [70]. CD105 is required for angiogenesis [71] and is regulated during heart development [72]. Since MSCs are known to promote angiogenesis [73, 74], perhaps the increase in CD105 expression is indicative of an increase in angiogenic potential. It has been shown that gene expression data of cell surface markers is representative of protein expression measured in flow cytometry [31]; thus, quantitating gene expression could be useful to estimate the percentage of cells expressing certain markers, when the use of antibodies prove to be difficult due to lack of availability and cross-reactivity in certain species, such as the goat. Nevertheless, there is still a need for antibodies with specificity to goats, as flow cytometry can provide information on marker expression on a per cell basis and has the potential to reflect heterogeneity in the MSC population.

Expression of CD34, a negative cell surface marker for MSCs defined by Dominici et al. [5] was detected in 9003 ASC at passage 5. This is consistent with a joint statement of the International Federation for Adipose Therapeutics and Science (IFATS) and the International Society for Cellular Therapy (ISCT) that CD34 expression in adipose-derived MSCs is variable and generally detected at early passages [10]. This could be due to heterogeneity in the early phase of ASC culture, as ASCs have been found to be more homogeneous in later passages [75]. This is consistent with the high standard deviation seen in the CFE of 9003 ASCs (which was due to differences between biological replicates, not technical replicates), suggesting a greater heterogeneity in the cell line. Detection of CD34 expression in early passage ASCs due to contamination of hematopoietic cells is also possible, and more population doublings in culture may be needed to attain homogeneity [63]. Another possible explanation is that CD34 is gradually lost in ASCs in *ex vivo* culture [75, 76].

In this study, the expression of the pluripotency-associated transcription factor genes *OCT4*, *SOX2* and *NANOG* were detected in our goat MSCs by RT-PCR, and OCT4 protein was detected by immunofluorescence. In the immunofluorescence experiment, OCT4 was shown to be localized exclusively in the cytoplasm of MSCs. This is consistent with the OCT4B isoform, which is the isoform associated with cell stress response [77, 78] and has been shown to be cytoplasmic [43, 79]. Localization of OCT4A, the isoform that is associated with pluripotency, is restricted to the nuclei of pluripotent cells [43, 79]. Cytoplasmic localization has been demonstrated in other reports of OCT4 detection in MSCs [29, 80, 81], confirming that the OCT4 isoform detected in MSCs is most likely OCT4B. A study on goat umbilical cord MSCs, which were reported to be more primitive, showed that OCT4 and SOX2 expression was more restricted to the nucleus with some expression in the cytoplasm, but with NANOG expression completely restricted to the cytoplasm [22]. When working with antibodies, one has to consider the possibility of non-specific staining, especially when using antibodies specific to other species and when no positive or negative control was used. One study reported that the detection of OCT4 and SOX2 expression in the nucleus and the cytoplasm varied with the different commercial antibodies used [82], demonstrating the questionable nature of the detection of these proteins in adult stem cells and the importance of using controls. In our study, we showed that the antibody used against OCT4 is specific to the nucleus in a bovine blastocyst, suggesting that the antibody was not isoform specific, and thus could be used to show differing localization that correlates with different isoforms. We also tried an antibody against NANOG in our study, which cross-reacted in our MSCs, but showed non-specific reactivity in bovine blastocysts (data not shown). *OCT4*, *NANOG* and *SOX2* were also expressed in the fibroblast line 1014 EF; it is then likely that these genes perform a different function in adult cells that is unrelated to pluripotency, or no function at all. This is supported by the observation that knocking out *OCT4* in MSCs as well as other somatic stem cells did not affect their self-renewal and multipotential abilities [83].

Investigating transfection and integration of introduced DNA constructs in MSCs is interesting for assessing their potential use in genetic engineering. The integration efficiencies observed in our goat MSCs are similar to the low efficiencies of random integration seen in other mammalian cells [84]. Haleem-Smith et al. saw no significant differences in transfection efficiency of human BM-MSCs from different donors [85], consistent with our findings. Nevertheless, they demonstrated higher Nucleofection efficiencies compared to ours, which could be explained by our use of a different plasmid [85], as well as the fact that the kits and protocols available were optimized for human MSCs and not goat MSCs. We also observed that, compared to 9004 BM-MSC, 9003 BM-MSC yielded significantly fewer integrant colonies and also lower CFE. This is unsurprising, as the subpopulation of cells that can attach and proliferate from low-density plating should represent the cells that are robust enough to stably integrate introduced DNA constructs, survive selection and proliferate into colonies of integrants.

Long-term *ex vivo* expansion of goat BM-MSCs may also have altered the proportion of MSC subpopulations. Higher passage BM-MSCs contained more enlarged, flattened and polygonal cells, which could be indicative of an increase in the large, flat and polygonal Type II subpopulation of MSCs [86, 87], which proliferate slower and tend to increase in proportion in higher passage MSCs [86, 88]. A large number of the lipid-filled adipocytes in P15 and P20 MSCs are rounded and appear less attached to the substrate, suggesting that the flatter cells may be less structurally supportive to adjacent cells. Additionally, a progressive decrease in G418-resistant integrant colonies from transfections was observed, even though DNA uptake ability of MSCs did not change with passaging. This suggests that genetic modification of higher passage MSCs may be less efficient due the increase of in the slow-growing Type II subpopulation that may be less robust against selection procedures. MSCs have been genetically modified *in vitro* for various applications, such as enhancing tissue regeneration [89, 90] or utilizing MSCs as delivery vehicles for transgene products [91–93]. In exploring the application of in cell-based therapy and tissue engineering, it is useful to investigate whether *ex vivo* expansion alters their amenability to genetic modification.

Our results show that two different adipogenic differentiation protocols resulted in significantly different profiles of adipogenesis, as shown by the morphology and percentage of Oil Red O-positive cells. MSCs that were exposed to alternating induction and maintenance media produced larger fat droplets than those produced by MSCs that were exposed to the same adipogenic medium consistently. The different patterns of adipogenesis due to different induction method show that MSCs could potentially be used as an *in vitro* model for illustrating adipogenesis [94]. There have been studies conducted on more differentiated cell types, such as immortalized pre-adipocytic cell lines or mature adipocytes from the stromal-vascular fraction in digested adipose tissue, as *in vitro* models to elucidate mechanisms involved in adipogenesis [95, 96]. Using adult stem cells like MSCs could bring another dimension to these studies; for instance, the mechanisms of differentiation and adipogenic lineage commitment could be examined as well.

Measuring adipogenic capacity by counting adipocytes is laborious, but is more informative as it takes into account the number of cells that actually differentiated. Methods that measure adipogenesis by solubilizing dyes and measuring absorbance do not provide information on the number of adipocytes or the average levels of lipids per cell. On the other hand, counting cells provides information on the number of cells that actually differentiated, and may be a better representation of adipogenic capacity and has been employed in other studies as well [25]. As demonstrated by our results, different methods of differentiation may yield more Oil Red O staining that do not necessarily equal to more adipocytes. It has been established that *FABP4* is one of the best markers of adipogenesis due to its significant upregulation after adipogenic induction [97]. Moreover, our results showed that *FABP4* expression is highly correlative to the number of lipid-positive cells in a given treatment. The large standard deviation seen in 9003 ASC’s *FABP4* expression (but not in *PPARG*), which was due to a large difference between the two biological replicates, mirrors the large standard deviation seen in the adipocyte count as well. The expression of *FABP4* (but not *PPARG*) in 9003 BM-MSC was also concordant with the adipocyte count, demonstrating that *FABP4* expression could be a reliable representation of adipocyte count. Hence, measuring *FABP4* mRNA could be used as quick measurement of the extent of adipogenesis within a given cell line. It was also shown in another study that semi-quantitative scoring of lipid staining was highly correlative to FABP4 protein quantification via flow cytometry, which also provides information on a per cell basis [48]. Results from our study and others [48] showed that *PPARG* expression was not representative of the extent of adipogenic differentiation, suggesting that expression of the early marker *PPARG* is not indicative of the progression of adipogenic differentiation.

## Conclusions

Our experiments demonstrate that 9004 BM-MSC, 9003 BM-MSC and 9003 ASC are indeed goat MSCs, as evidenced by their ability to undergo osteogenic, chondrogenic and adipogenic differentiation. Like human MSCs, goat MSCs also express CD90, CD105 and CD73, and do not express the hematopoietic marker CD45. As evidenced by CFE, different MSC isolations from the same tissue type can result in significantly different quantities of progenitor cells. Thus, performing a colony-forming assay early in a MSC culture may be useful to assess the purity of a MSC isolate and influence choice of cell lines for future experiments.

This study also sheds light on how the donor, source tissue and passage number of goat MSCs could lead to differences in adipogenic differentiation. Cytochemical staining and gene expression analysis showed that the goat ASC line had a greater capacity for adipogenic differentiation compared to the two BM-MSC lines. Goat fibroblasts were also able to undergo adipogenic differentiation, but at a low capacity. The differences in adipogenic potential between two BM-MSC lines demonstrate donor-to-donor variability between MSCs isolated from the same tissue, even when the donors were siblings from the same litter. Last but not least, passaging and prolonged *ex vivo* culture of BM-MSCs had an impact on adipogenicity as well.

In conclusion, this study provides further characterization information on goat MSCs, as well as an insight to how MSC characteristics change during *ex vivo* expansion. BM-MSCs expanded over increasing passages *in vitro* maintained karyotypic stability up to 20 passages in culture, exhibited an increase in adipogenic differentiation and CFE, but showed altered morphology and amenability to genetic modification by selection. Different methods of inducing adipogenesis also affect the extent and profile of adipogenic differentiation in MSCs, indicating the potential of goat MSCs as an *in vitro* model for adipogenesis for ruminant species.

## Electronic supplementary material

Additional file 1: **This PDF file contains Supplementary Figures 1, 2 and 3, as mentioned in the manuscript text, along with the materials and methods involved to produce these figures.** (PDF 2 MB)

## Abbreviations

ASCs: Adipose-derived mesenchymal stem cells
BM-MSCs: Bone marrow-derived mesenchymal stem cells
CFE: Colony-forming efficiency
CFU: Colony-forming unit
FBS: fetal bovine serum
EGFP: Enhanced green fluorescent protein
MSCs: Mesenchymal stem cells
Neo^R^: Neomycin resistance/resistant
P5: Passage 5
P10: Passage 10
P15: Passage 15
P20: Passage 20
PBS: Phosphate-buffered saline.
