# Supplementary material for: Impact of source tissue and ex vivo expansion on the characterization of goat mesenchymal stem cells
Source: J Anim Sci Biotechnol. 2015 Jan 11;6(1):1. doi: 10.1186/2049-1891-6-1 (PMC4382838; doi:10.1186/2049-1891-6-1)
Supplement: Supplementary file 1 — Additional file 1: This PDF file contains Supplementary Figures 1, 2 and 3, as mentioned in the manuscript text, along with the materials and methods involved to produce these figures. (PDF 2 MB) [file 40104_2014_139_MOESM1_ESM.pdf]

Supplementary Methods and Figures

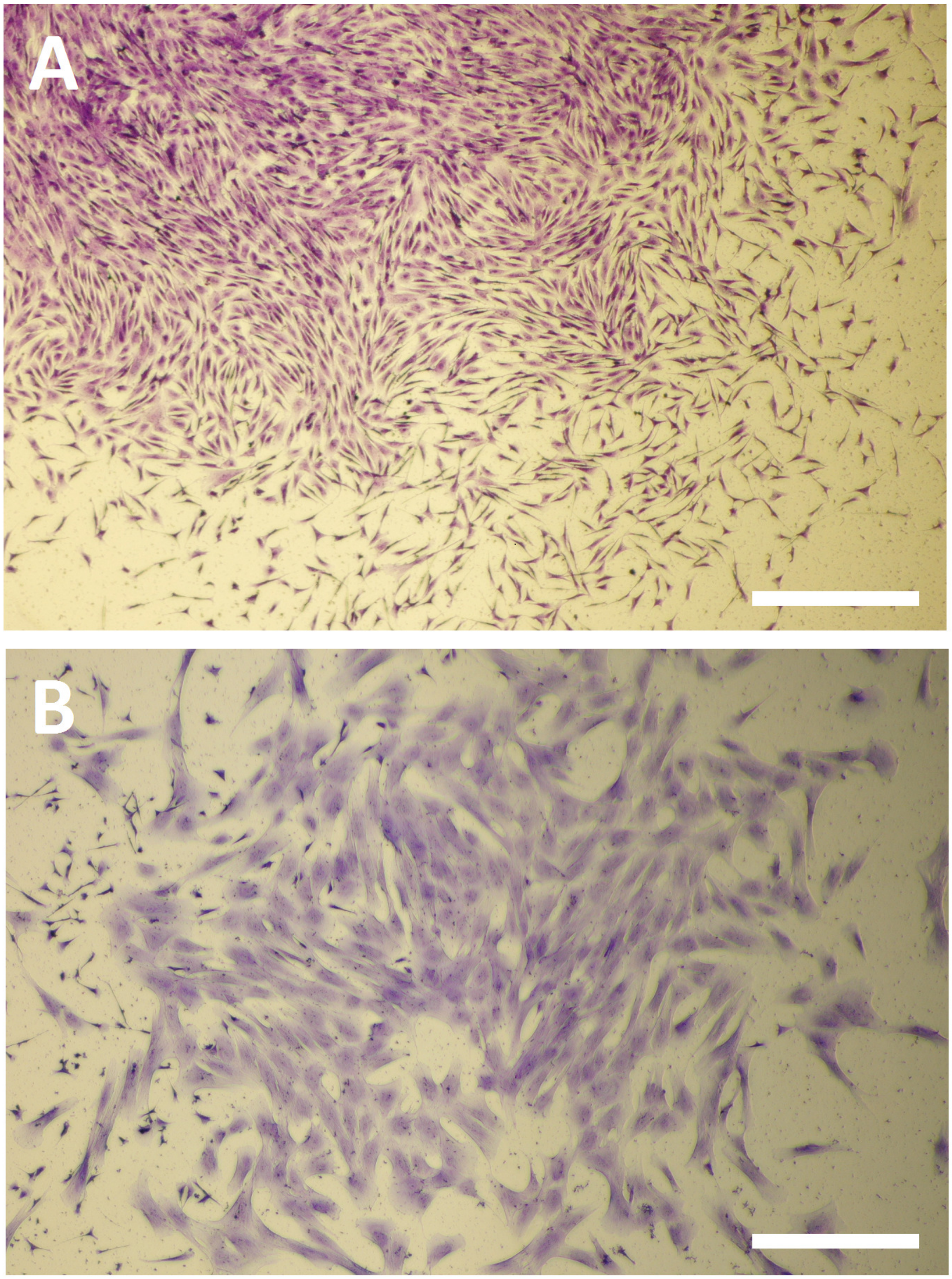

Figure S1. Different types of MSC colonies from colony-forming unit assay. Representative images of 9004 BM-MSC colonies are shown in phase contrast at 40X magnification: A) big colony of smaller, proliferative cells, and B) smaller colony of larger, flatter cells that are slower in proliferation. Scale bars represent 500 µm.

Methods for Figures S2 and S3

Passage 5 9004 BM-MSC and 9003 BM-MSC were plated in 6-well plates at a density of 10,000 cells/cm^2 and cultured to 80% confluency. Then, expansion medium was replaced with adipogenic induction medium: DMEM (Gibco Life Technologies 12100-046), 10% FBS (JR Scientific), 1.0 µM dexamethasone (Sigma), 0.5 mM 3-isobutyl-1-methylxanthine (Sigma), 200 µM indomethacin (Sigma) and 10 µM insulin (Akron Biotech). Cells were cultured for 10 days with medium changes every 3 days. Control cells were cultured in expansion medium instead of induction medium for the duration of the experiment.

At the end of the assay, cells were fixed and stained with Oil Red O as described in Materials and Methods. To quantify adipogenic differentiation capacity, images of cells were captured to represent at least 4,000 cells per replicate of each cell line. Lipid-positive cells that stained with Oil Red O were counted using ImageJ (National Institutes of Health). Adipocyte count is expressed as the percentage of lipid-positive cells over the total number of nuclei counted. This assay was done in triplicates and the experiment repeated once more.

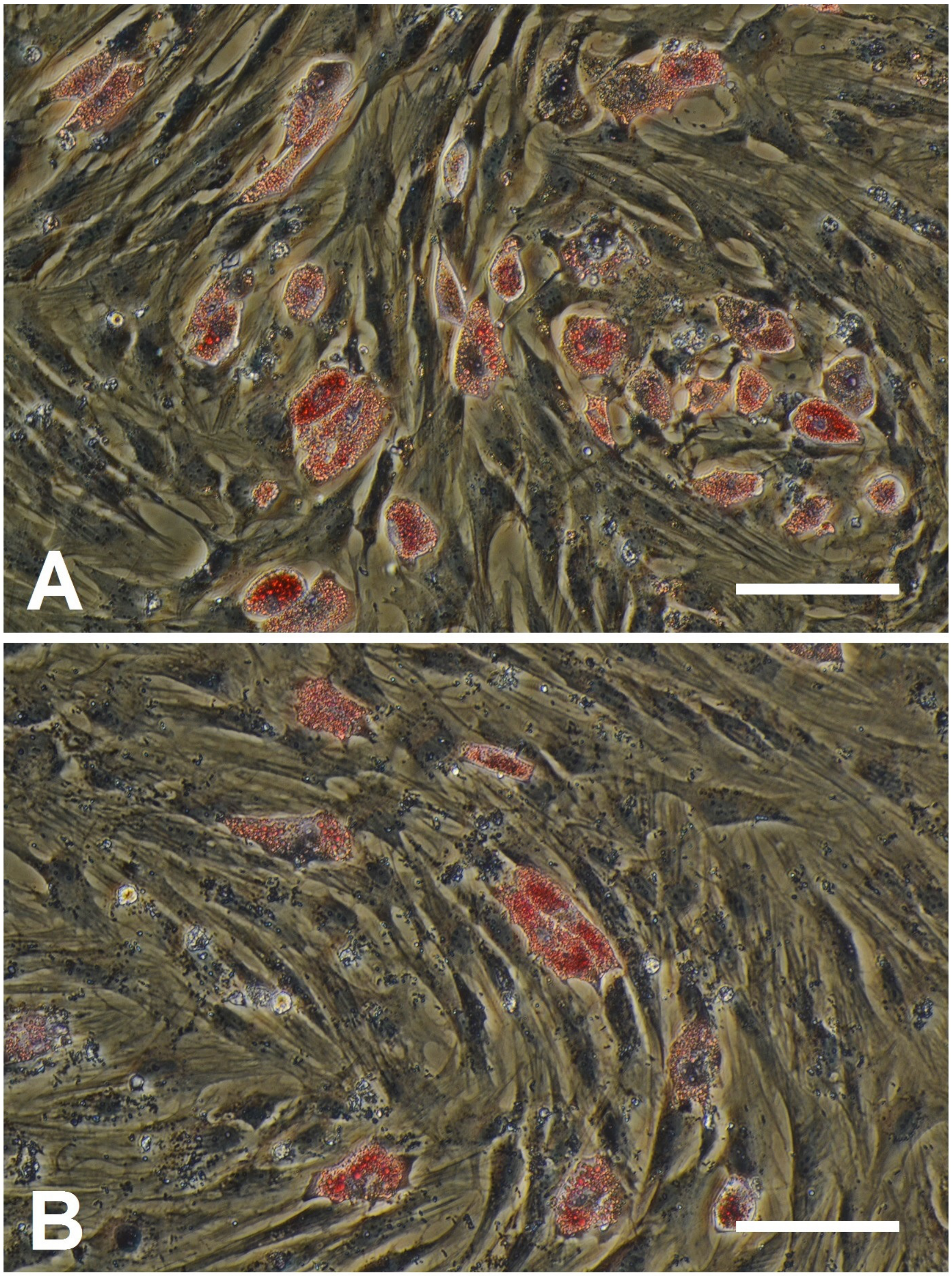

Figure S2. Oil Red O staining of BM-MSCs after 10 days of induction. 9004 BM-MSC (A) and 9003 BM-MSC (B) were cultured in adipogenic induction medium for 10 days and stained with Oil Red O. Differentiated adipocytes accumulated lipid droplets in the cytoplasm that stain red. Representative images are shown in phase contrast at 200X magnification. Scale bars represent 100 μm.

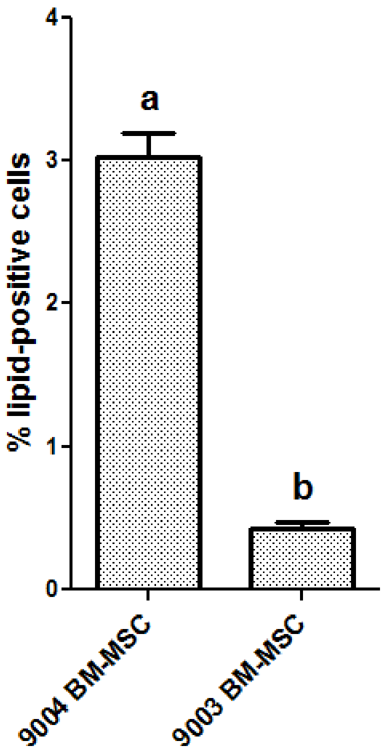

Figure S3: Proportion of adipocytes in BM-MSCs after 10 days of induction. The percentage of lipid-positive cells over total cells counted (± SEM) is shown for 9004 BM-MSC and 9003 BM-MSC differentiated for 10 days. Statistically significant groups determined by Tukey's multiple means comparison test are indicated by letters ($p < 0.05$).
